# Demographic, regional and temporal trends of hyperuricemia epidemics in mainland China from 2000 to 2019: a systematic review and meta-analysis

**DOI:** 10.1080/16549716.2021.1874652

**Published:** 2021-01-21

**Authors:** Yang Li, Ziyan Shen, Bowen Zhu, Han Zhang, Xiaoyan Zhang, Xiaoqiang Ding

**Affiliations:** a Department of Nephrology, Zhongshan Hospital, Fudan University, Shanghai, China; b Shanghai Medical Center of Kidney, Shanghai, China; c Shanghai Key Laboratory of Kidney and Blood Purification, Shanghai, China; d Shanghai Institute of Kidney and Dialysis, Shanghai, China; e Hemodialysis Quality Control Center of Shanghai, China

**Keywords:** Hyperuricemia, China, epidemiology, systematic review, meta-analysis

## Abstract

**Background:**

Hyperuricemia (HUA) is becoming a global public health problem and associated with multiple diseases.

**Objective:**

We conducted a systematic review to synthesize the pooled prevalence of HUA in mainland China and delineate its demographic, regional, and temporal trends over the past two decades.

**Methods:**

Systematic literature searches of PubMed, SCOPUS, Web of Science, the China National Knowledge Infrastructure (CNKI), and the Wanfang digital database were conducted to screen studies published from 1 January 2000 to 31 August 2019, reporting the prevalence of HUA in mainland China. The search strings were (‘hyperuricemia’ OR ‘hyperuricaemia’ OR ‘uric acid’) AND (‘prevalence’ OR ‘epidemiology’) AND ‘China’. Article quality was appraised quantitatively from 11 items. Before formal meta-analysis, age-standardized prevalence was transformed. The random-effects model was applied to synthesize the pooled prevalence due to its high heterogeneity. Then we stratified the prevalence estimates by age, gender, area, nationality, and publication year for subgroup analysis.

**Results:**

Totally 177 eligible studies with a whole population of 2,277,712 were included in the present meta-analysis. The pooled prevalence in mainland China was estimated at 16.4% (95% CI: 15.3%~17.6%). In studies with the onset age at 20 ~ 29 years old, males had a twice times higher HUA prevalence than females (21.5% vs. 8.9%). The prevalence of HUA was 13.7% (11.8%~15.7%) in people aged 15~ years old, 16.5% in 30~ (14.8%~18.4%), 17.9% in 40~ (16.4%~19.5%), 19.4% in 50~ (17.8%~21.0%), 20.5% in 60~ (18.8%~22.3%), and 24.9% in over 70 (22.9%~27.1%). Stratified by regions, southern (25.5%) and southwestern (21.2%) China shared the highest prevalence, and the lowest prevalence was observed in the northwest (12.6%). From 2001 to 2017, the prevalence estimates of HUA steadily climbed from 8.5% to 18.4% with minor fluctuations. Multiple regression revealed that HUA prevalence was positively correlated to the larger sample size, area, advanced onset age, and published year.

**Conclusions:**

The last two decades witnessed the rapid growth prevalence of HUA in China. Early screening and personalized health education for HUA need to be given enough attention.

## Background

Hyperuricemia (HUA), characterized as a metabolic disease of purine disorders, is becoming a global public health problem. Traditional recognition of HUA was limited to its triggering of gout and related disability-adjusted of life years [1]. Recently, more studies have proved that HUA was also associated with cardiovascular diseases, renal dysfunction, and cancers [2–4]. For each increase of 1 mg/dL in serum uric acid (SUA) level, the pooled relative risk of stroke and coronary heart disease mortality was 1.10 and 1.13, respectively [5]. With socioeconomic development, gout incidence has more than doubled worldwide in the past two decades [6]. Meanwhile, the prevalence of HUA also keeps growing in many developed countries. In Italy, HUA prevalence increased from 8.5% in 2005 to 11.9% in 2009 [7]. Another longitudinal study in Australia revealed a significant increase in SUA both in men and women from 1959 to 1980 [8].

China is the largest developing country. 1.4 billion Chinese people are spreading in 34 regions with the varied socioeconomic, cultural, and behavioral background. Since the geographic characteristics are widely divergent, few surveys on HUA were conducted in a cross-regional scope [9,10]. Moreover, the pathophysiologic of HUA is complex. Increased uric acid production and/or decreased renal excretion capacity could result in HUA. Specific dietary patterns and alcohol use contributed to the diverse prevalence of HUA in different regions. Hence, understanding the epidemics of HUA and its social factors is essential to estimate the disease burden in China. With this information, targeted and personalized anti-hyperuricemia healthcare can be implemented.

To this end, we carried out a systematic review to synthesize the pooled prevalence of HUA in mainland China and to delineate the demographic, regional, and temporal distribution over the past two decades.

## Methods

### Literature search strategy

The systematic literature search was performed in PubMed, SCOPUS, Web of Science, the China National Knowledge Infrastructure (CNKI), and the Wanfang digital database. We updated the search on 31 August 2019, and limited the language to English and Chinese. For Pubmed database, the search strings were ((uric acid [MeSH Terms]) OR (hyperuricemia [MeSH Terms])) AND ((epidemiology [Title/Abstract]) OR (prevalence [Title/Abstract])) AND (China [Title/Abstract] OR (Chinese [Title/Abstract])) AND (‘2000/01/01’[Date – Publication]: ‘2019/08/31’[Date – Publication]) AND (English [Language]). Then we use the filter panel to select studies with full text. For SCOPUS database, the search strings were (TITLE-ABS (‘uric acid’ OR ‘Hyperuricemia’ OR ‘hyperuricaemia’) AND TITLE-ABS (‘prevalence’ OR ‘epidemiology’) AND TITLE-ABS (‘China’) AND LANGUAGE (English)) AND DOCTYPE (ar) AND PUBYEAR > 1999 AND PUBYEAR < 2020. For Web of Science database, the search strings were (TI = (uric acid) OR TI = (hyperuricemia) OR TI = (hyperuricaemia) OR AK = (uric acid) OR AK = (hyperuricemia) OR AK = (hyperuricaemia)) AND (TI = (prevalence) OR AB = (prevalence) OR TI = (epidemiology) OR AB = (epidemiology)) AND (TI = (China) OR AB = (China) OR TS = (China)) AND (LANGUAGE = (English) AND (Indexes = SCI-EXPANDED, SSCI, A&HCI, CPCI-S, CPCI-SSH, ESCI, CCR-EXPANDED) AND (IC Timespan = 2000–2019). Similarly, Chinese-language literature was searched, from CNKI and Wanfang database, by using the medical subject heading terms and/or keywords including ‘hyperuricemia’, ‘uric acid’, ‘prevalence’, and ‘epidemiology’. In addition, we also searched the related references for the screened literature if necessary.

### Inclusion and exclusion criteria

The criteria of literature screening were as follows: (1) original study conducted in mainland China; (2) community-based cross-sectional study design; (3) the published time was during 1 January 2000 and 31 August 2019; (4) the onset age of survey was ≥15 years old, including both males and females; (5) age-specific prevalence of HUA was reported; (6) sample size ≥ 1000; (7) with detailed information on the participants’ demographic, HUA diagnostic criteria and sampling process. We further excluded studies that were hospital-based, combined with other diseases, animal experiments, duplicated, case-control or intervention studies, conference abstracts or reviews, diagnostic criteria lacking or not classified by gender, and not available for age standardization.

### Study selection

Literature was imported into Endnote X9 software. Firstly, the title and abstract were screened independently by two researchers (Y.L. and Z.Y.S.). Articles that did not met the above eligible criteria of (1)(2)(3) were excluded from this process. Then we downloaded the full text and reviewed them for compliance with the remaining criteria. Any disagreements were referred to the third researcher (X.Y.Z.) for judgment to avoid selection bias.

### Data extraction and data items

We extracted the necessary data items for meta-analysis. It included first author, published year, survey time, age range, survey region, HUA diagnostic criteria (male/female), sample size (male/female), cases (male/female), age-adjusted prevalence (male/female), sampling method, sample source, and study design, etc.

### Quality appraisal

The quality assessment was performed simultaneously according to the ‘cross-sectional or prevalence study quality’ checklist [11]. It was designed by the U.S. Agency for Healthcare Research and Quality in 2004, initially to evaluate the researches on celiac disease. This checklist had 11 items, including data source, inclusion criteria, survey time, community-based population, avoiding selective bias, measurement quality assessment, explaining the reasons for exclusion, potential confounding control, dealing with missing data, participant response rate, follow-up conditions. Each qualified item was awarded one point out of eleven. More detailed descriptions of this checklist could be seen in Supplementary Table 1.

### HUA diagnosis and classification

HUA was defined as a fasting serum uric acid level >420 μmol/L in males and >360 μmol/L in females measured on two separate days after a regular purine diet [12]. Each diagnostic criterion was listed in Supplementary Table 2. Due to the geographic and economic disparities, we classified the 31 mainland provinces, municipalities and autonomous regions in mainland China as the eastern, northern, central, southern, southwestern, northwestern, and northeastern regions of China. According to the onset age, the study population was divided into five groups: 15~, 20~, 30~, 40~, and 50~. Other subgroups were analyzed in gender (male vs. female), nationality (Han vs. minority), sample size (<4999 vs. ≥5000), published year (2001 ~ 2005, 2006 ~ 2010, 2011 ~ 2015 and 2016 ~ 2019), and age-specific group (15~, 30~, 40~, 50~, 60~, 70~).

### Statistical analysis

The fifth and sixth China National Population Census of 2000 and 2010 provided the overall population data by 5-year age group and gender [13,14]. We applied these data to calculate the standardized prevalence of HUA in each literature, for reducing the demographic differences in studies. Meta-analysis was performed in ‘meta’ packages of R program 3.6.0 (R core team). Firstly, the Freeman-Tukey double arcsine transformation was utilized to convert the prevalence rates into a quantity. It made them satisfy a normal distribution and suitable for either the fixed- or random-effect model. The heterogeneity of the studies was evaluated by the Chi-squared-based Q test and *I^2^* test. The *I^2^* values of 25%, 50%, and 75% referred to low, moderate, and high heterogeneity, respectively. The pooled prevalence estimates were calculated in a fixed-effect model (if *I^2^ *< 25% or p > 0.05 in Q test) or a random-effect model (if *I^2^ *> 25% or p < 0.05 in Q test). Subgroup analysis was applied to delineate the demographic, regional, and temporal trends of HUA prevalence. In addition, meta-regression analysis were conducted to identify the major contributors to high heterogeneity. The potential publication bias was assessed by using the Begg’s test and Egger’s test. The significant level of α was set at 0.05, and all tests were two-tailed.

## Results

### Basic characteristics and quality appraisal of enrolled studies

**Figure 1.**
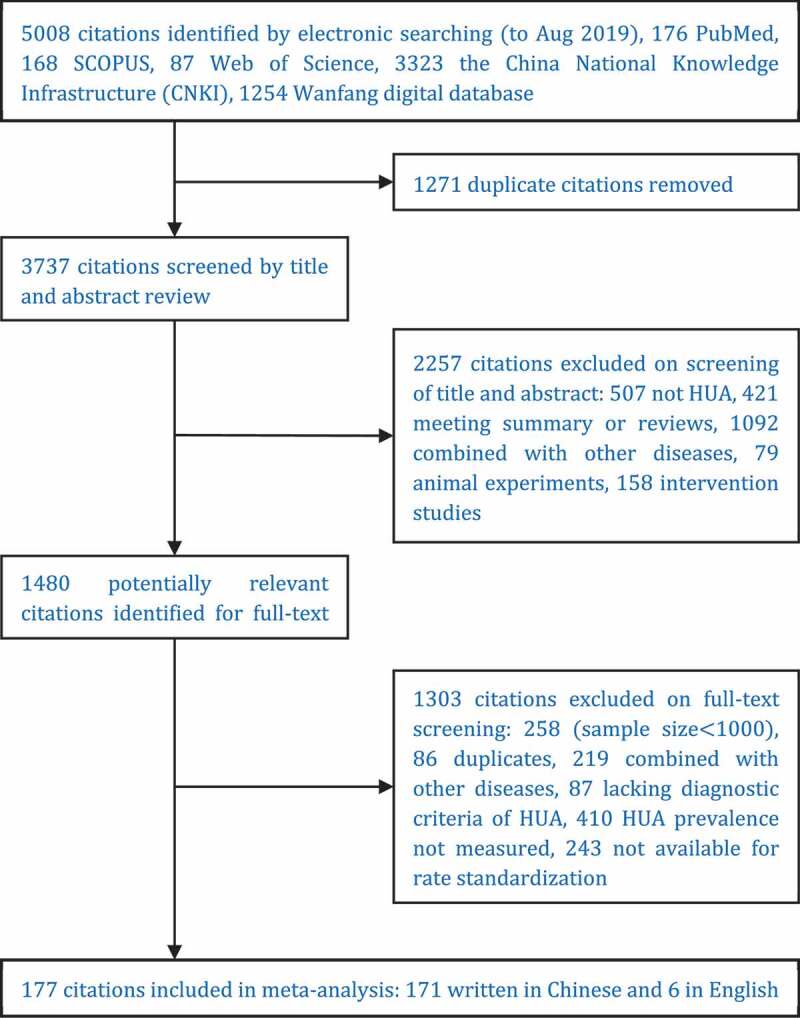
Flow diagram for literature screening

Figure 1 detailed the flow diagram of literature search. A total of 5,008 citations were initially identified under the search strategies. After two-stage study selection, 177 studies met the inclusion criteria (171 written in Chinese and 6 in English). It enrolled 2,277,712 participants, of which 1,304,353 were male (57.3%) and 973,359 were female (42.7%). Classified by the onset age of study, these articles were divided into five groups: 15~(N = 51, n = 1,099,992), 20~(N = 96, n = 1,041,320), 30~(N = 11, n = 46,758), 40~(N = 9, n = 33,762), 50~(N = 10, n = 55,880).

The detailed characteristics of 177 studies were shown in Supplementary Tables 2–4 and Supplementary Text 1. All studies were cross-sectional, conducted in community population (n = 51), physical examination population (n = 85), occupational population (n = 40), and college students (n = 1). Multistage randomized sampling was applied in nearly one-quarter of studies (n = 44). The response rate of 166 was over 90.0%. As for quality appraisal, the average score of eligible studies was 7.81 ± 1.07, with a maximum of 10 and a minimum of 6. Of them, fifty-seven studies scored 8 (32.2%), thirty-four studies scored 9 (19.2%), and twelve studies scored 10 (6.8%).

### Pooled age-standardized prevalence of hyperuricemia

**Figure 2.**
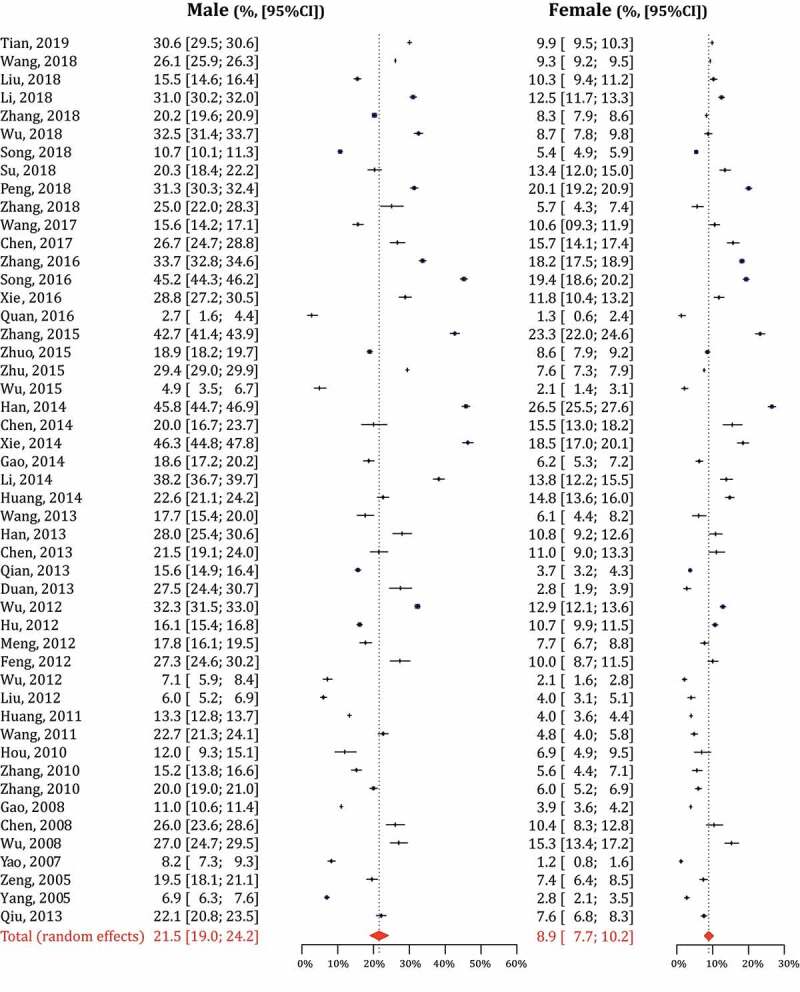
Forest plot of hyperuricemia prevalence for study initiated from 20 years old

**Figure 3.**
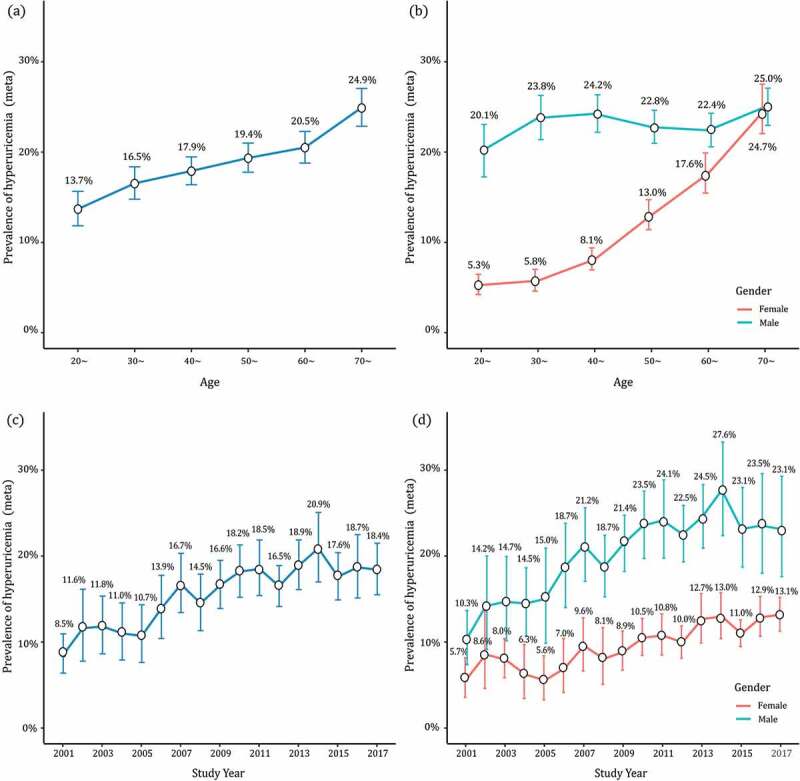
Demographic and temporal trends of hyperuricemia prevalence

The age-standardized prevalence of HUA ranged from 1.8% to 42.2%, with a pooled prevalence of 16.4% (95% CI: 15.3%~17.6%). The subgroup analysis of pooled HUA prevalence was shown in Table 1, including gender, nationality, sample size, area, published year, etc. The Han Chinese had a higher HUA prevalence than ethnic-minority people (16.6% vs. 13.4%). In studies with the onset age at 20~ years old (Figure 2), the prevalence of HUA in males (21.5%, 95% CI: 19.0%~24.2%) was twice as high as that in females (8.9%, 95% CI: 7.7%~10.2%). Moreover, the prevalence also kept increasing with age (Figure 3(a)). The prevalence of HUA was 13.7% (95% CI: 11.8%~15.7%) in participants aged 15 ~ 29 years old, and 16.5% in 30~ (95% CI: 14.8%~18.4%), 17.9% in 40~ (95% CI: 16.4%~19.5%), 19.4% in 50~ (95% CI: 17.8%~21.0%), 20.5% in 60~ (95% CI: 18.8%~22.3%), and 24.9% in over 70 (95% CI: 22.9%~27.1%). Stratified by gender, the age-specific prevalence of HUA in males increased to the first peak in the group of 40 ~ 49 (24.2%), then maintained a plateau between 50 ~ 69 (22.4%~22.8%) until the second peak in 70~ (25.0%). While, in females, this estimate was on the continuous rise with age. And the prevalence in females increased sharply from 50 years old, and also peaked in 70~ (24.7%), with the gender margin shrunk to a similar level (Figure 3(b)).

**Table 1.** Prevalence of hyperuricemia according to different categories

					Heterogeneity test		Publication bias	
Category	Category	No. of Studies	Prevalence (95% CI, %)	No. of Participants	I^2^ (%)	p _(Q Test)_	p_(Begg’s Test)_	p(_Egger’s Test)_
Total	Total	177	16.4 (15.3 ~ 17.6)	2,277,712	99.8	<0.001	0.755	0.872
Nationality	Han	168	16.6 (15.5 ~ 17.8)	2,244,635	99.8	<0.001	0.888	0.976
	Minority	9	13.4 (7.2 ~ 21.1)	33,077	99.7	<0.001	0.345	0.516
Gender	Male	177	20.4 (18.9 ~ 22.0)	1,304,353	99.8	<0.001	0.776	0.121
	Female	177	9.8 (8.9 ~ 10.8)	973,359	99.6	<0.001	0.445	0.942
Sample size	<4999	81	15.4 (13.9 ~ 17.1)	209,998	99.0	<0.001	0.987	0.802
	≥5000	96	17.3 (15.7 ~ 18.9)	2,067,714	99.9	<0.001	0.752	0.616
Onset age (yr)	15 ~ 19	51	15.6 (13.8 ~ 17.4)	1,099,992	99.8	<0.001	0.622	0.679
	20 ~ 39	96	17.1 (15.4 ~ 18.9)	1,041,320	99.8	<0.001	0.560	0.942
	30 ~ 49	11	12.1 (8.4 ~ 16.3)	46,758	99.4	<0.001	0.815	0.281
	40 ~ 59	9	18.6 (12.4 ~ 25.7)	33,762	99.6	<0.001	1.000	0.307
	50~	10	18.6 (16.4 ~ 21.0)	55,880	97.8	<0.001	0.929	0.774
Age-specific (yr)	15 ~ 29	89	13.7 (11.8 ~ 15.7)	194,668	99.3	<0.001	0.323	0.330
	30 ~ 39	103	16.5 (14.8 ~ 18.4)	331,839	99.5	<0.001	0.880	0.454
	40 ~ 49	116	17.9 (16.4 ~ 19.5)	352,469	99.3	<0.001	0.852	0.865
	50 ~ 59	116	19.4 (17.8 ~ 21.0)	256,954	99.1	<0.001	0.894	0.618
	60 ~ 69	108	20.5 (18.8 ~ 22.3)	166,876	98.7	<0.001	0.793	0.160
	70~	78	24.9 (22.9 ~ 27.1)	89,529	98.0	<0.001	0.759	0.103
Region	Eastern	52	14.3 (12.4 ~ 16.2)	768,298	99.8	<0.001	0.463	0.903
	Northern	28	13.8 (12.3 ~ 15.4)	707,593	99.6	<0.001	0.693	0.031
	Central	7	13.7 (10.3 ~ 17.4)	75,173	99.4	<0.001	0.881	0.656
	Southern	31	25.5 (22.1 ~ 29.0)	361,537	99.8	<0.001	0.377	0.946
	Southwestern	19	21.2 (17.4 ~ 25.3)	170,770	99.7	<0.001	0.327	0.513
	Northwestern	30	12.6 (10.4 ~ 15.0)	129,336	99.3	<0.001	0.205	0.114
	Northeastern	9	15.9 (13.0 ~ 19.0)	28,657	97.8	<0.001	0.532	0.440
Published year	2001 ~ 2005	4	11.3 (7.1 ~ 16.5)	34,942	99.5	<0.001	1.000	0.718
	2006 ~ 2010	41	14.7 (12.0 ~ 17.7)	316,903	99.8	<0.001	0.459	0.109
	2011 ~ 2015	81	16.6 (14.9 ~ 18.4)	1,009,523	99.8	<0.001	0.674	0.487
	2016 ~ 2019	51	18.0 (16.0 ~ 20.0)	916,344	99.8	<0.001	0.329	0.517

### Prevalence of hyperuricemia in the seven regions of mainland China

**Figure 4.**
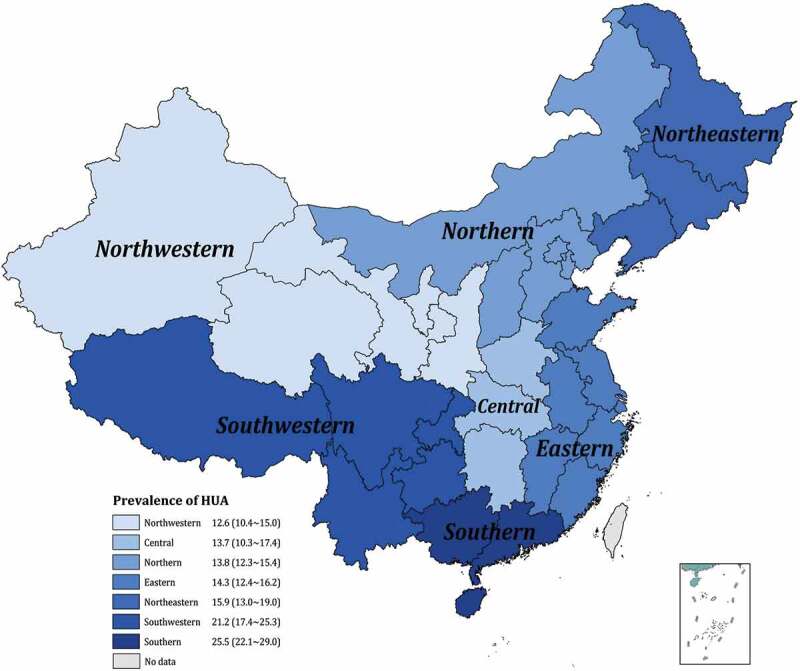
Regional distribution of hyperuricemia prevalence in mainland China

To better understand the geographical distribution of HUA, we further sub-analyzed the regional prevalence in the seven areas of China: the eastern, northern, central, southern, southwestern, northwestern, and northeastern (Figure 4). The pooled prevalence of HUA maintained the highest level in the southern (25.5%, 95% CI: 22.1%~29.0%) and the southwestern (21.2%, 95% CI: 17.4%~25.3%), statistically higher than that in the rest regions (all p-value < 0.001). The moderate prevalence was in the northeastern (15.9%, 95% CI: 13.0%~19.0%) and eastern (14.3%, 95% CI: 12.4%~16.2%). Participants in northern and central areas shared a comparable prevalence between 13.8% (95% CI: 12.3%~15.4%) and 13.7% (95% CI: 10.3%~17.4%). The lowest HUA prevalence was observed in northwestern China (12.6%, 95% CI: 10.4%~15.0%).

### Temporal trends of hyperuricemia from 2001 to 2017

The survey time in this meta-analysis spanned seventeen years (2001 ~ 2017), with the prevalence of HUA steadily climbed to over two times of that in 2001. As shown in Figure 3(c), the prevalence of HUA was on the rise before 2014, and the estimate increased from 8.5% (95% CI: 6.4%~10.9%) to 20.9% (95% CI: 17.0%~25.1%). It began to decline slowly and rebounded to a level of 18.4% (95% CI: 15.5%~21.5%) in 2017. Stratified by gender, such a similar trend of ‘rise-fall-rebound’ was also found in males: the prevalence increased initially from 10.3% (95% CI: 7.4%~13.7%) in 2001 to 27.6% (95% CI: 22.3%~33.2%) in 2014, and exhibited a slight decrease until 2017 (23.1%, 95% CI: 17.6%~29.2%). In female participants, the trend of HUA prevalence maintained an upward rise during the survey time: from 5.7% (95% CI: 3.6%~8.2%) in 2001 to 13.2% (95% CI: 11.3%~15.2%) in 2017 (Figure 3(d)).

### Analysis of heterogeneity and publication bias

The overall heterogeneity between studies was noted as a significantly high level (*I^2^* = 99.8%, p < 0.001); even in subgroup analysis, the heterogeneity only decreased slightly. Hence, we performed the meta-regression to identify the major contributors to high heterogeneity (Table 2). Univariate analysis revealed that the HUA prevalence increased 16.4% and 11.3% in participants from the southern and southeastern as compared with those from northwestern China. The higher the article quality score gained, the lower the HUA prevalence was (β = −0.022, p = 0.002). In multiple regression, such a positive correlation was also found in studies with larger sample size, advanced onset age (50~), and published year since 2006. Egger’s linear regression test and Begg’s test showed that no significant publication bias was in both overall and subgroup analysis (Table 1).

**Table 2.** Univariate and multivariable meta-regression for hyperuricemia prevalence

		Univariate analysis		Multivariable analysis	
Category	Subgroup	Coefficient (95% CI)	p-value	Coefficient (95% CI)	p-value
Sex ratio	Continuous	0.007(−0.004 ~ 0.018)	0.224	0.005(−0.006 ~ 0.016)	0.389
Sample size	<4999 vs ≥5000	0.026(−0.004 ~ 0.057)	0.087	0.036(0.003 ~ 0.068)	0.032
Region	Northwestern vs Central	0.013(−0.063 ~ 0.090)	0.731	0.024(−0.055 ~ 0.102)	0.552
	Northwestern vs Northern	0.015(−0.033 ~ 0.064)	0.533	0.012(−0.040 ~ 0.064)	0.659
	Northwestern vs Eastern	0.022(−0.020 ~ 0.064)	0.300	0.025(−0.021 ~ 0.071)	0.281
	Northwestern vs Southwestern	0.113(0.060 ~ 0.166)	<0.001	0.112(0.055 ~ 0.168)	<0.001
	Northwestern vs Northeastern	0.045(−0.025 ~ 0.114)	0.207	0.084(0.012 ~ 0.156)	0.025
	Northwestern vs Southern	0.164(0.117 ~ 0.210)	<0.001	0.176(0.126 ~ 0.226)	<0.001
Nationality	Han vs Other	−0.046(−0.115 ~ 0.023)	0.189	−0.027(−0.095 ~ 0.042)	0.444
Onset age	15 ~ 19 vs 20 ~ 25	0.021(−0.015 ~ 0.057)	0.256	0.028(−0.006 ~ 0.063)	0.110
	15 ~ 19 vs 30 ~ 35	−0.051(−0.118 ~ 0.018)	0.151	0.000(−0.065 ~ 0.065)	0.995
	15 ~ 19 vs 40 ~ 45	0.041(−0.034 ~ 0.115)	0.284	0.058(−0.011 ~ 0.128)	0.100
	15 ~ 19 vs 50 ~ 65	0.041(−0.030 ~ 0.112)	0.262	0.078(0.011 ~ 0.144)	0.023
Published year	2001 ~ 2005 vs 2006 ~ 2010	0.051(−0.058 ~ 0.159)	0.359	0.098(−0.001 ~ 0.196)	0.050
	2001 ~ 2005 vs 2011 ~ 2015	0.078(−0.027 ~ 0.184)	0.146	0.114(0.019 ~ 0.209)	0.019
	2001 ~ 2005 vs 2016 ~ 2019	0.094(−0.013 ~ 0.201)	0.084	0.139(0.042 ~ 0.236)	0.005
Response rate	Continuous	0.262(−0.064 ~ 0.587)	0.115	−0.092(−0.424 ~ 0.241)	0.588
Quality score	Continuous	−0.022(−0.036~-0.008)	0.002	−0.015(−0.031 ~ 0.001)	0.055

## Discussion

It is the first study attempting to synthesize the pooled standardized prevalence estimations of HUA among Chinese populations. Through a systematic literature search, we enrolled 177 articles (2,277,712 participants) to delineate the epidemiological distribution of HUA in mainland China over the past 20 years. The age-standardized prevalence was transformed before meta-analysis to minimize the differences of demographic characteristics between studies. In studies with the onset age at 20 ~ 29 years old, the HUA prevalence in males and females were 21.5% and 8.9%, confirming HUA a considerable disease burden in China. Moreover, the prevalence also kept increasing from 13.7% in 15 ~ 29 years old to 24.9% in the over 70 years old. It may be explained by the physiological reduction of the renal glomerular filtration rate with aging. SUA is the end product of purine metabolism, generated by nucleic acids and other purine compounds produced by catabolism of systematic nuclear proteins and food intake. Over two-thirds of SUA is excreted through the kidney, filtrated 100% in glomerulus, retaken 90% in proximal tubule, and the rest discharged [15]. The glomerular filtration rate has been proved to physiologically decline 0.75 ~ 1 ml/min with healthy aging in humans after 40 years old [16]. However, this trend of gender differs moderately. In males, the pooled prevalence was high along with the all adult age, peaked in the groups of 40 ~ 49 and 70 ~ . Unlike hypertension or type 2 diabetes, HUA is also commonly encountered in the young and middle-aged, which could be explained by the food impact on SUA level [17,18]. About 20% of SUA daily generated is from food intake, while the barbecue and hot pot with purine-rich food are getting popular in the young [19]. Moreover, males tend to indulge in alcohol and sugared beverages than females, and these drinks have been proved to interfere with uric acid metabolism and increase the risk of HUA [20,21]. This trend indicates that health education and screening should be conducted in young males. While, in females, the prevalence of HUA maintained relatively low from 5.3% to 8.1%, but increased sharply from 50 years old, till similar to that of males in 70 ~ . Such a trend is associated with menopause. Estrogen inhibits the biosynthesis and promotes the excretion of SUA, and the estrogen in females before menopause is 2 ~ 3 times of males of the same age [22]. Elevated body mass index, insulin resistance, and alcohol ingestion may also be the factors contributing to the increased prevalence of HUA in postmenopausal females [23]. Thus, in females after menopause, the awareness of HUA should also be enhanced.

The distribution of HUA also showed geographical differences. The prevalence was higher in southern China (25.5%), southwestern China (21.2%), and northeastern China (15.9%), which may be attributed to regional eating habit, either is rich in purine content or interferences with the metabolism of purine. In southern, seafood is among the daily diets, while the hot pot is the most popular in southwestern. Alcohol is most consumed in northeast China than in the rest regions. In our study, HUA’s extremum prevalence was observed in Guangdong (42.2%) and Xinjiang (1.8%). Minority localities and limited sample size in border areas of China may contribute to the extremely low prevalence of Xinjiang, indicating a need for studies with a larger sample size and gene polymorphisms in these areas. The relatively poor awareness rate, treatment of HUA in these areas also should be paid attention to. Health education should be expanded to cover these populations, and regional specified dietary instruction should be focused on high-risk groups.

It was observed that, in the past two decades, the prevalence of HUA in mainland China doubled with a steady increase rate in both males and females. Augmented life pressure, shifted diet patterns and habits, reduced physical exercise are all potential factors contributing to this phenomenon [19,24–28]. Given the huge population base in China, this doubled prevalence has tremendous implications for public health. Intensified interventions must be taken now to deal with this situation, which was neglected before. Apart from active treatment, prevention measures such as health education should be taken immediately at the community level, which can profoundly impact the whole population.

This meta-analysis estimated the age-standardized prevalence of HUA and analyzed the demographic, regional, and temporal distribution in mainland China throughout the past 20 years. Still, there are some limitations needed to be clarified. Firstly, high heterogeneity was noted in subgroup analysis, due to the vast geographic area and publication time span. We performed meta-regression to identify the major contributors to high heterogeneity. Surveys focusing on the southern and southeastern regions, larger sample size, advanced onset age, and published year were associated with higher HUA prevalence. Secondly, data of other factors may influence heterogeneity and partly contribute to the HUA prevalence (e.g., dietary patterns, alcohol consumption, stressful life, sedentary lifestyle, etc.) were not complete in the reviewed studies. Further large-scale studies should take these aspects into account. Thirdly, no longitudinal study was found in literature screening, and the included studies are almost cross-sectional design, which can only provide the prevalence but not infer the causal sequence.

To conclude, the last 20 years witnessed a double prevalence of HUA in mainland China with steady growth. Young and middle-aged males, residents in south and southwest China need to be given enough attention to with early and personalized education for HUA, and the elderly also should be screened and treated in time.

## Supplementary Material

Supplemental Material

## Paper context

It is the first study attempting to synthesize the pooled standardized prevalence estimations of hyperuricemia among Chinese populations. The last 20 years witnessed a rapid growth of hyperuricemia prevalence in mainland China. Early screening and personalized health education for hyperuricemia is needed.
