# Supplementary material for: Demographic, regional and temporal trends of hyperuricemia epidemics in mainland China from 2000 to 2019: a systematic review and meta-analysis
Source: Glob Health Action. 2021 Jan 21;14(1):1874652. doi: 10.1080/16549716.2021.1874652 (PMC7833047; doi:10.1080/16549716.2021.1874652)
Supplement: Supplemental Material [file ZGHA_A_1874652_SM6560.docx]

**Supplementary Table 1. Cross-sectional/prevalence study quality**

| **Item** | **Yes** | **No** | **Unclear** |
| --- | --- | --- | --- |
| 1) Define the source of information (survey, record review) |  |  |  |
| 2) List inclusion and exclusion criteria for exposed and unexposed subjects (cases and controls) or refer to previous publications |  |  |  |
| 3) Indicate time period used for identifying patients |  |  |  |
| 4) Indicate whether or not subjects were consecutive if not population-based |  |  |  |
| 5) Indicate if evaluators of subjective components of study were masked to other aspects of the status of the participants |  |  |  |
| 6) Describe any assessments undertaken for quality assurance purposes (e.g., test/retest of primary outcome measurements) |  |  |  |
| 7) Explain any patient exclusions from analysis |  |  |  |
| 8) Describe how confounding was assessed and/or controlled. |  |  |  |
| 9) If applicable, explain how missing data were handled in the analysis |  |  |  |
| 10) Summarize patient response rates and completeness of data collection |  |  |  |
| 11) Clarify what follow-up, if any, was expected and the percentage of patients for which incomplete data or follow-up was obtained |  |  |  |

**Copyright:**

Rostom A, Dubé C, Cranney A, et al. Celiac Disease: Evidence Reports/Technology Assessments, No. 104. Appendix D. Quality Assessment Forms. Agency for Healthcare Research and Quality (US); 2004. Available: <http://www.ncbi.nlm.nih.Gov/books>. NBK35156. Bookshelf ID: NBK35156, Accessed 2020 Feb 5.

**Supplementary Table 2. Summary of studies on the prevalence of hyperuricemia**

| **No.** | **First author (published year)** | **Survey Time** | **Age range** | **Survey region** | **Area** | **Diagnostic criterion (μmol/L) (M/F)** | **Sample size (M/F)** | **HUA no. (M/F)** | **HUA (%)*** | **HUA (%)* (M/F)** |
| --- | --- | --- | --- | --- | --- | --- | --- | --- | --- | --- |
| 1 | Ai et al. (2019) | Jan. 2017~Dec. 2017 | 20~ | Xinjiang | Northwestern | ≥420/≥360 | 3579/2197 | 629/203 | 14.5 | 17.7/10.0 |
| 2 | Pu et al. (2019) | Jan. 2018~Dec. 2018 | 21~98 | Jiangsu | Eastern | ≥428/≥357 | 1914/1534 | 441/141 | 17.1 | 25.0/8.5 |
| 3 | Chu et al. (2019) | Jan. 2017~Dec. 2017 | 65~ | Shanghai | Eastern | ≥421/≥359 | 1590/1776 | 311/428 | 20.6 | 19.2/21.9 |
| 4 | Wei et al. (2019) | Jan. 2016~Dec. 2017 | 65~93 | Shanghai | Eastern | ≥420/≥360 | 901/1146 | 207/291 | 24.7 | 23.2/25.9 |
| 5 | Tian et al. (2019) | Jan. 2017~Dec. 2017 | 18~ | Beijing | Northern | ≥420/≥360 | 27419/25254 | 8524/2271 | 20.3 | 30.1/9.9 |
| 6 | Zhang et al. (2019) | Jan. 2016~Dec. 2016 | 20~83 | Guizhou | Southwestern | ≥420/≥360 | 662/1229 | 263/386 | 30.8 | 44.6/24.5 |
| 7 | Li et al. (2018) | Mar. 2016~Oct. 2016 | 45~ | Ningxia | Northwestern | ≥420/≥357 | 2241/903 | 411/96 | 16.3 | 17.7/14.0 |
| 8 | Zhao et al. (2018) | Mar. 2016~Jul. 2016 | 20~93 | Anhui | Eastern | ≥428/≥357 | 1131/1420 | 229/141 | 14.5 | 20.6/9.7 |
| 9 | Wang et al. (2018) | Jan. 2012~Dec. 2017 | 18~ | Beijing | Northern | ≥416/≥357 | 206881/192208 | 52915/16356 | 17.7 | 26.1/9.3 |
| 10 | Liu et al. (2018) | Jan. 2003~Dec. 2005 | 6~99 | Guangxi | Southern | ≥420/≥360 | 5812/4265 | 1062/377 | 13.3 | 15.5/10.3 |
| 11 | Huang et al. (2018) | Jan. 2016~Dec. 2016 | 20~90 | Guangdong | Southern | ≥420/≥360 | 15105/8480 | 7747/2453 | 42.2 | 52.4/24.9 |
| 12 | Li et al. (2018) | May. 2016~May. 2017 | 16~93 | Hebei | Northern | ≥428/≥357 | 10324/6949 | 2931/894 | 23.0 | 31.0/12.5 |
| 13 | Zhang et al. (2018) | Jul. 2015~Sept. 2017 | 18~79 | Henan | Central | ≥417/≥357 | 15371/23484 | 1968/2010 | 12.6 | 20.2/8.3 |
| 14 | Wu et al. (2018) | Jan. 2015~Dec. 2015 | 16~87 | Hainan | Southern | ≥420/≥320 | 6171/2996 | 1697/197 | 23.6 | 32.5/8.8 |
| 15 | Song et al. (2018) | Jan. 2016~Dec. 2016 | 18~82 | Guangdong | Southern | ≥420/≥350 | 10223/8216 | 1293/521 | 8.3 | 10.7/5.4 |
| 16 | Zhang et al. (2018) | Jan. 2015~Dec. 2015 | 20~94 | Ningxia | Northwestern | ≥420/≥350 | 12115/7241 | 3180/700 | 20.3 | 26.2/10.7 |
| 17 | Su et al. (2018) | Mar. 2013~May. 2013 | 18~ | Zhejiang | Eastern | ≥420/≥350 | 1797/2108 | 330/364 | 16.4 | 20.3/13.4 |
| 18 | Peng et al. (2018) | Jun. 2017~Dec. 2017 | 15~85 | Shanghai | Eastern | ≥420/≥360 | 8010/8541 | 2522/1906 | 25.4 | 31.3/20.1 |
| 19 | Zhang et al. (2018) | Jan. 2015~Dec. 2016 | 17~86 | Xinjiang | Northwestern | ≥420/≥360 | 759/918 | 169/42 | 13.8 | 25.0/5.6 |
| 20 | Wu et al. (2017) | Oct. 2014~Jun. 2016 | 40~ | Xinjiang | Northwestern | ≥420/≥350 | 711/823 | 102/101 | 12.4 | 12.8/12.3 |
| 21 | He et al. (2017) | May. 2014~Nov. 2014 | 22~91 | Liaoning | Northeastern | ≥420/≥350 | 1044/958 | 252/106 | 26.3 | 37.9/13.7 |
| 22 | Liu et al. (2017) | Jan. 2016~Dec. 2016 | 20~90 | Shanxi | Northwestern | ≥420/≥360 | 442/587 | 101/58 | 14.1 | 22.0/8.8 |
| 23 | Yan et al. (2017) | Jan. 2016~Dec. 2016 | 20~ | Gansu | Northwestern | ≥420/≥350 | 4101/2238 | 881/43 | 15.6 | 22.9/1.7 |
| 24 | Bian et al. (2017) | May. 2016~Nov. 2016 | 20~ | Jiangsu | Eastern | ≥420/≥350 | 2064/1284 | 513/184 | 18.4 | 11.3/25.2 |
| 25 | Wang et al. (2017) | Jan. 2014~Dec. 2015 | 18~ | Zhejiang | Eastern | ≥420/≥360 | 2464/2132 | 538/136 | 12.3 | 15.6/10.5 |
| 26 | Jiang et al. (2017) | Jan. 2012~Dec. 2015 | 20~89 | Yunnan | Southwestern | ≥416/≥339 | 2058/1827 | 833/490 | 29.0 | 39.1/18.1 |
| 27 | Kong et al. (2017) | Jan. 2010~Jan. 2016 | 20~88 | Guangdong | Southern | ≥420/≥350 | 6500/3550 | 2968/886 | 38.3 | 46.2/25.5 |
| 28 | Feng et al. (2017) | Jan. 2016~Dec. 2016 | 20~ | Jiangsu | Eastern | ≥420/≥360 | 2260/787 | 341/67 | 13.2 | 14.5/9.1 |
| 29 | Guo et al. (2017) | Jun. 2015~Aug. 2015 | 21~80 | Ningxia | Northwestern | ≥420/≥357 | 660/500 | 159/26 | 16.2 | 24.0/5.5 |
| 30 | Chen et al. (2017) | May. 2016~Dec. 2016 | 18~89 | Guangxi | Southern | ≥420/≥350 | 1818/1965 | 489/307 | 21.0 | 26.7/15.7 |
| 31 | Liu et al. (2017) | Jan. 2015~Dec. 2015 | 65~ | Shanghai | Eastern | ≥420/≥350 | 2455/3346 | 515/642 | 20.0 | 20.9/19.3 |
| 32 | Han et al. (2017) | Aug. 2012~Jul. 2013 | 20~74 | Shandong | Eastern | ≥420/≥360 | 5059/3373 | 1183/247 | 16.2 | 20.7/6.7 |
| 33 | Wang et al. (2017) | Jan. 2012~Dec. 2014 | 25~75 | Xinjiang | Northwestern | ≥420/≥360 | 4798/5534 | 854/603 | 13.8 | 18.0/10.4 |
| 34 | Zhang et al. (2017) | Mar. 2006~Nov. 2006 | 25~60 | Ningxia | Northwestern | ≥417/≥357 | 3967/1488 | 1319/195 | 21.9 | 25.5/13.1 |
| 35 | Xie et al. (2017) | Jan. 2015~Dec. 2015 | 35~75 | Guangdong | Southern | ≥417/≥357 | 1410/1177 | 175/104 | 10.6 | 12.5/8.2 |
| 36 | He et al. (2017) | May. 2012~May. 2013 | 25~80 | Hunan | Central | ≥420/≥350 | 1433/1528 | 540/130 | 19.2 | 31.3/7.8 |
| 37 | Dan et al. (2016) | Nov. 2014~Oct. 2015 | 23~81 | Anhui | Eastern | ≥420/≥350 | 706/570 | 114/33 | 10.2 | 14.4/5.0 |
| 38 | Zhang et al. (2016) | Jan. 2015~Dec. 2015 | 20~93 | Shanxi | Northern | ≥417/≥339 | 3968/2751 | 714/308 | 15.5 | 18.6/11.1 |
| 39 | Wang et al. (2016) | Jan. 2009~Dec. 2009 | 55~ | Beijing | Northern | ≥420/≥357 | 652/805 | 115/192 | 20.1 | 17.6/22.3 |
| 40 | Wang et al. (2016) | Jan. 2000~Dec. 2000 | 55~ | Beijing | Northern | ≥420/≥357 | 1098/1093 | 106/107 | 9.6 | 10.0/9.3 |
| 41 | Zhang et al. (2016) | Jan. 2012~Dec. 2014 | 18~95 | Guangdong | Southern | ≥420/≥350 | 10132/11330 | 3174/1303 | 25.6 | 33.7/18.2 |
| 42 | Li et al. (2016) | Aug. 2014~Aug. 2015 | 20~85 | Guangdong | Southern | ≥420/≥350 | 1252/588 | 572/104 | 34.1 | 41.9/17.4 |
| 43 | Song et al. (2016) | Jan. 2014~Dec. 2015 | 3~94 | Guangdong | Southern | ≥420/≥350 | 10722/9560 | 4904/1886 | 33.3 | 45.2/19.3 |
| 44 | Xie et al. (2016) | Jul. 2013~Dec. 2013 | 18~61 | Beijing | Northern | ≥420/≥357 | 2859/2161 | 768/157 | 22.5 | 28.8/11.8 |
| 45 | Sheng et al. (2016) | Jan. 2014~Dec. 2014 | 45~ | Gansu | Northwestern | ≥420/≥420 | 1400/760 | 216/78 | 13.1 | 15.1/9.6 |
| 46 | Zhang et al. (2016) | Jan. 2013~Dec. 2013 | 20~59 | Shandong | Eastern | ≥420/≥357 | 4550/1274 | 907/47 | 16.3 | 20.3/4.3 |
| 47 | Quan et al. (2016) | Oct. 2015~Dec. 2015 | 18~97 | Xinjiang | Northwestern | ≥420/≥350 | 592/710 | 16/9 | 1.8 | 2.7/1.2 |
| 48 | Shen et al. (2016) | Jan. 2012~Dec. 2012 | 20~ | —— | Northwestern | ≥420/≥357 | 3014/1166 | 649/46 | 17.0 | 21.5/4.1 |
| 49 | Li et al. (2016) | Jan. 2010~Dec. 2014 | 20~ | Chongqing | Southwestern | ≥420/≥357 | 18139/7928 | 1272/324 | 6.8 | 9.6/5.4 |
| 50 | Liu et al. (2015) | Jan. 2013~Dec. 2013 | 20~ | Guangdong | Southern | ≥420/≥360 | 2257/1980 | 859/475 | 29.9 | 36.5/22.3 |
| 51 | Zhou et al. (2015) | Jun. 2013~Aug. 2013 | 20~55 | Henan | Central | ≥417/≥357 | 4869/933 | 1214/89 | 22.2 | 24.5/8.9 |
| 52 | Jiang et al. (2015) | Jan. 2015~Jun. 2015 | 21~86 | Fujian | Eastern | ≥428/≥357 | 26325/24731 | 6756/2698 | 18.9 | 25.7/11.8 |
| 53 | Lv et al. (2015) | Jan. 2014~May. 2014 | 20~91 | Yunnan | Southwestern | ≥420/≥350 | 11664/8109 | 4242/416 | 24.5 | 37.1/6.2 |
| 54 | Zhang et al. (2015) | Jan. 2013~Dec. 2013 | 21~71 | Gansu | Northwestern | ≥416/≥339 | 2791/2571 | 362/124 | 8.4 | 11.7/4.6 |
| 55 | Zhou et al. (2015) | Jan. 2011~Dec. 2012 | 20~60 | Anhui | Eastern | ≥420/≥350 | 22085/7241 | 3390/352 | 12.8 | 15.7/5.2 |
| 56 | Fan et al. (2015) | Aug. 2011~Dec. 2012 | 45~ | Ningxia | Northwestern | ≥420/≥350 | 634/661 | 113/29 | 12.2 | 18.7/4.5 |
| 57 | Li et al. (2015) | Jan. 2013~Dec. 2013 | 40~ | Guangdong | Southern | ≥420/≥350 | 3634/5355 | 1584/1879 | 37.9 | 33.2/44.3 |
| 58 | Lin et al. (2015) | Jan. 2013~Dec. 2013 | 25~ | Zhejiang | Eastern | ≥416/≥357 | 3702/1679 | 216/35 | 5.3 | 6.1/5.0 |
| 59 | Zhang et al. (2015) | Jan. 2014~Dec. 2014 | 18~ | Guangdong | Southern | ≥420/≥357 | 5991/4110 | 2568/874 | 33.5 | 42.7/23.2 |
| 60 | Zhuo et al. (2015) | Jul. 2008~Jul. 2010 | 18~ | Jiangsu | Eastern | ≥420/≥360 | 10413/6737 | 2072/800 | 15.1 | 18.9/8.6 |
| 61 | Li et al. (2015) | Dec. 2010~Sept. 2014 | 20~ | Guangxi | Southern | ≥417/≥357 | 27144/24062 | 10722/3459 | 27.9 | 39.2/15.9 |
| 62 | Liu et al. (2015) | Oct. 2013~Dec. 2013 | 20~93 | Anhui | Eastern | ≥428/≥357 | 2792/2101 | 435/202 | 11.9 | 16.7/6.8 |
| 63 | Zhu et al. (2015) | Jan. 2011~Dec. 2013 | 10~98 | Fujian | Eastern | ≥416/≥357 | 38533/28019 | 11003/1671 | 20.1 | 29.4/7.6 |
| 64 | Mei et al. (2015) | Jan. 2012~Dec. 2014 | 20~65 | Shanxi | Northern | ≥420/≥360 | 3051/2145 | 918/76 | 11.0 | 11.9/9.6 |
| 65 | Wu et al. (2015) | Dec. 2008~Jan. 2009 | 16~96 | Xinjiang | Northwestern | ≥420/≥350 | 735/1186 | 35/22 | 3.2 | 4.9/2.1 |
| 66 | Luo et al. (2015) | Jan. 2010~Dec. 2012 | 20~70 | Hubei | Central | ≥420/≥357 | 3284/3166 | 505/119 | 9.6 | 15.2/3.2 |
| 67 | Dong et al. (2015) | Jan. 2013~Dec. 2013 | 25~ | Inner Mongolia | Northern | ≥420/≥360 | 829/870 | 212/28 | 13.8 | 26.7/3.1 |
| 68 | Han et al. (2014) | Jan. 2006~Dec. 2006 | 23~85 | Jiangsu | Eastern | ≥420/≥360 | 7565/3176 | 2074/244 | 20.9 | 27.8/8.1 |
| 69 | Han et al. (2014) | Jan. 2011~Dec. 2011 | 23~85 | Jiangsu | Eastern | ≥420/≥360 | 7565/3176 | 2501/346 | 25.3 | 33.5/10.5 |
| 70 | Zhang et al. (2014) | Jan. 2011~Dec. 2012 | 30~69 | Zhejiang | Eastern | ≥420/≥350 | 7548/5668 | 1758/509 | 18.5 | 23.7/11.6 |
| 71 | Cui et al. (2014) | Jan. 2012~Dec. 2012 | 20~94 | Hebei | Northern | ≥420/≥350 | 9833/9919 | 1968/648 | 13.4 | 20.8/8.1 |
| 72 | Han et al. (2014) | Jan. 2012~Dec. 2013 | 18~89 | Guangdong | Southern | ≥428/≥357 | 8442/7190 | 3885/1780 | 37.1 | 45.8/26.5 |
| 73 | Yang et al. (2014) | Jan. 2009~Dec. 2012 | 30~78 | Shanxi | Northwestern | ≥420/≥357 | 1060/844 | 235/151 | 17.1 | 22.5/12.8 |
| 74 | Chen et al. (2014) | Sept. 2002~Dec. 2002 | 18~ | Guangxi | Southern | ≥420/≥357 | 525/763 | 109/123 | 17.2 | 19.9/15.5 |
| 75 | Song et al. (2014) | Jun. 2011~Oct. 2011 | 40~ | Jiangxi | Eastern | ≥420/≥350 | 1824/1971 | 488/307 | 22.6 | 27.8/17.5 |
| 76 | Qiang et al. (2014) | Jan. 2007~Dec. 2009 | 20~78 | Ningxia | Northwestern | ≥420/≥357 | 3318/2097 | 736/172 | 16.6 | 21.6/9.9 |
| 77 | Cao et al. (2014) | Jan. 2012~Dec. 2012 | 20~ | Shandong | Eastern | ≥420/≥360 | 8399/2835 | 1589/179 | 15.6 | 19.3/5.8 |
| 78 | Xie et al. (2014) | Jan. 2011~Dec. 2011 | 18~60 | Guangdong | Southern | ≥420/≥360 | 4453/2442 | 2085/471 | 35.2 | 46.3/18.5 |
| 79 | Gao et al. (2014) | Jan. 2012~Jun. 2012 | 18~97 | Shanxi | Northwestern | ≥420/≥357 | 2731/2649 | 492/193 | 12.6 | 18.6/6.2 |
| 80 | Li et al. (2014) | Jan. 2009~Dec. 2011 | 18~ | Tibet | Southwestern | ≥420/≥357 | 3988/1703 | 1533/222 | 30.9 | 38.2/13.8 |
| 81 | Zhang et al. (2014) | Dec. 2012~Mar. 2013 | 30~92 | Hebei | Northern | ≥420/≥350 | 982/1127 | 94/83 | 8.2 | 9.6/7.0 |
| 82 | Liu et al. (2014) | Jan. 2009~Dec. 2010 | 60~89 | Jiangsu | Eastern | ≥420/≥357 | 4591/2713 | 668/320 | 15.4 | 17.0/13.0 |
| 83 | Huang et al. (2014) | Sept. 2007~Jun. 2009 | 18~75 | Guangxi | Southern | ≥420/≥360 | 2779/3494 | 789/688 | 18.2 | 22.6/14.8 |
| 84 | Wang et al. (2013) | Jan. 2012~Dec. 2012 | 18~79 | Jiangsu | Eastern | ≥428/≥357 | 1088/660 | 194/34 | 13.1 | 17.6/6.1 |
| 85 | Han et al. (2013) | Sept. 2011~Dec. 2011 | 19~ | Heilongjiang | Northeastern | ≥420/≥350 | 1159/1277 | 311/142 | 18.6 | 28.0/10.8 |
| 86 | Zhao et al. (2013) | Jan. 2011~Dec. 2012 | 21~89 | Hebei | Northern | ≥417/≥357 | 6332/2781 | 1388/295 | 20.2 | 24.9/8.9 |
| 87 | Chen et al. (2013) | Jan. 2010~Dec. 2010 | 18~ | Gansu | Northwestern | ≥417/≥357 | 1151/844 | 240/126 | 17.1 | 21.4/11.0 |
| 88 | Liu et al. (2013) | Jan. 2012~Dec. 2012 | 20~90 | Hebei | Northern | ≥420/≥357 | 30835/17069 | 4307/750 | 9.5 | 12.6/3.9 |
| 89 | Yang et al. (2013) | Apr. 2012~Jun. 2012 | 40~ | Shanghai | Eastern | ≥408/≥357 | 3912/5359 | 879/880 | 14.3 | 18.7/11.5 |
| 90 | Guo et al. (2013) | Jan. 2010~Dec. 2010 | 20~86 | Heilongjiang | Northeastern | ≥420/≥350 | 1756/1524 | 305/125 | 11.9 | 16.6/6.5 |
| 91 | Sun et al. (2013) | Jan. 2012~Dec. 2012 | 20~75 | Beijing | Northern | ≥420/≥360 | 698/393 | 103/4 | 9.3 | 13.7/0.4 |
| 92 | Zhao et al. (2013) | Jan. 2012~Dec. 2012 | 21~80 | Sichuan | Southwestern | ≥417/≥357 | 8524/4187 | 2570/466 | 23.9 | 30.6/12.9 |
| 93 | Jia et al. (2013) | Aug. 2010~Sept. 2011 | 20~88 | Hebei | Northern | ≥420/≥357 | 19807/14001 | 3947/1188 | 18.4 | 18.1/19.4 |
| 94 | Qian et al. (2013) | Mar. 2010~Sept. 2011 | 18~ | Hubei | Central | ≥420/≥360 | 8478/5566 | 1341/230 | 11.0 | 15.6/3.7 |
| 95 | Dong et al. (2013) | Mar. 2009~Aug. 2009 | 18~ | Xinjiang | Northwestern | ≥417/≥357 | 823/1223 | 228/33 | 11.9 | 27.5/2.8 |
| 96 | Wang et al. (2013) | Dec. 2010 | 20~70 | Guangdong | Southern | ≥420/≥357 | 1023/1077 | 420/160 | 27.6 | 38.8/17.0 |
| 97 | Song et al. (2012) | Jan. 2011~Dec. 2011 | 61~99 | Tianjin | Northern | ≥417/≥357 | 1431/1993 | 266/284 | 15.5 | 18.0/13.8 |
| 98 | Tan et al. (2012) | Jan. 2007~Dec. 2009 | 60~ | Sichuan | Southwestern | ≥420/≥357 | 2271/296 | 603/71 | 25.5 | 25.7/23.8 |
| 99 | Liu et al. (2012) | Jan. 2010~Jul. 2010 | 20~ | Sichuan | Southwestern | ≥417/≥357 | 24174/17327 | 6897/1462 | 20.0 | 28.5/9.4 |
| 100 | Wu et al. (2012) | Jan. 2009~Dec. 2009 | 6~84 | Guangdong | Southern | ≥417/≥357 | 15205/7790 | 4801/650 | 25.8 | 32.2/12.9 |
| 101 | Lu et al. (2012) | Jan. 2009~Dec. 2011 | 20~89 | Guizhou | Southwestern | ≥420/≥357 | 1929/686 | 477/74 | 20.6 | 23.7/12.4 |
| 102 | Hu et al. (2012) | Jan. 2011~Dec. 2011 | 18~96 | Zhejiang | Eastern | ≥420/≥360 | 10061/6450 | 1681/738 | 13.8 | 16.1/10.7 |
| 103 | Meng et al. (2012) | Jan. 2010~May. 2010 | 18~ | Jiangsu | Eastern | ≥420/≥360 | 2102/2402 | 331/207 | 11.4 | 17.8/7.7 |
| 104 | Feng et al. (2012) | Aug. 2011~Sept. 2011 | 16~42 | Yunnan | Southwestern | ≥420/≥350 | 1039/1895 | 253/204 | 17.9 | 27.4/10.0 |
| 105 | Yang et al. (2012) | Jun. 2009~Jun. 2010 | 20~90 | Ningxia | Northwestern | ≥420/≥350 | 2348/1526 | 468/197 | 16.5 | 20.2/10.4 |
| 106 | Wang et al. (2012) | Oct. 2007~Oct. 2010 | 23~63 | Beijing | Northern | ≥417/≥357 | 1570/102 | 304/9 | 19.1 | 19.9/9.2 |
| 107 | Wu et al. (2012) | Jan. 2008~Jun. 2008 | 14~96 | Xinjiang | Northwestern | ≥420/≥350 | 1745/2237 | 111/47 | 4.3 | 7.1/2.1 |
| 108 | She et al. (2012) | Jan. 2008~Dec. 2008 | 20~85 | Jiangsu | Eastern | ≥417/≥357 | 7226/3360 | 1190/189 | 12.4 | 15.8/5.3 |
| 109 | Liu et al. (2012) | Jan. 2010~Aug. 2010 | 18~97 | Henan | Central | ≥420/≥357 | 3213/1508 | 207/56 | 5.3 | 6.0/4.0 |
| 110 | Feng et al. (2012) | Jan. 2007~May. 2009 | 24~67 | Yunnan | Southwestern | ≥417/≥357 | 1874/993 | 646/136 | 28.2 | 34.9/15.7 |
| 111 | Wang et al. (2012) | Mar. 2009~Apr. 2011 | 21~63 | Xinjiang | Northwestern | ≥417/≥357 | 784/258 | 92/36 | 12.6 | 12.4/13.7 |
| 112 | Qian et al. (2011) | Jan. 2010~Dec. 2010 | 22~87 | Shanghai | Eastern | ≥420/≥357 | 2442/1024 | 199/105 | 8.6 | 8.1/10.4 |
| 113 | Wen et al. (2011) | Jan. 2010~Dec. 2010 | 20~90 | Guangxi | Southern | ≥420/≥350 | 4246/1565 | 1620/199 | 31.1 | 37.6/13.3 |
| 114 | Huang et al. (2011) | May. 2009~Apr. 2010 | 18~94 | Zhejiang | Eastern | ≥417/≥357 | 19399/9835 | 2733/280 | 10.1 | 13.3/4.0 |
| 115 | Liang et al. (2011) | Jul. 2008~Jul. 2009 | 21~95 | Guangdong | Southern | ≥420/≥350 | 1531/917 | 278/107 | 12.9 | 15.4/11.7 |
| 116 | Yang et al. (2011) | Apr. 2010~Jul. 2010 | 25~76 | Gansu | Northwestern | ≥420/≥350 | 862/791 | 150/86 | 13.8 | 16.9/10.8 |
| 117 | Liu et al. (2011) | Mar. 2009~Dec. 2009 | 22~93 | Jiangsu | Eastern | ≥428/≥340 | 5023/2742 | 768/132 | 11.4 | 15.4/4.9 |
| 118 | Zhang et al. (2011) | Jan. 2009~Nov. 2010 | 45~84 | Guizhou | Southwestern | ≥420/≥350 | 1441/979 | 387/102 | 17.2 | 22.2/9.3 |
| 119 | Huang et al. (2011) | Apr. 2009~Feb. 2010 | 20~ | Guangxi | Southern | ≥420/≥350 | 3267/2133 | 1562/521 | 38.5 | 47.9/25.6 |
| 120 | Chen et al. (2011) | Sept. 2010~Dec. 2010 | 30~83 | Guangdong | Southern | ≥420/≥350 | 1787/2085 | 753/421 | 29.6 | 42.3/18.5 |
| 121 | Zhu et al. (2011) | Jul. 2009~Dec. 2009 | 20~91 | Guangdong | Southern | ≥417/≥357 | 18832/14817 | 5892/2205 | 25.0 | 30.9/17.5 |
| 122 | Liu et al. (2011) | Jan. 2008~Dec. 2008 | 20~ | Shanxi | Northern | ≥417/≥357 | 5282/6338 | 578/520 | 8.5 | 10.0/7.2 |
| 123 | Wang et al. (2011) | Jun. 2009~Jun. 2010 | 16~90 | Liaoning | Northeastern | ≥420/≥350 | 3560/2323 | 758/107 | 15.1 | 22.7/4.8 |
| 124 | Liu et al. (2011) | Jan. 2008~Dec. 2009 | 20~93 | Tianjin | Northern | ≥420/≥350 | 16272/12389 | 2464/780 | 11.5 | 15.2/6.8 |
| 125 | Jia et al. (2011) | Jul. 2007~Jul. 2009 | 20~ | Tianjin | Northern | ≥420/≥360 | 10900/6841 | 1708/446 | 12.1 | 16.0/5.7 |
| 126 | Chen et al. (2011) | Jan. 2006~Dec. 2009 | 20~ | Jiangsu | Eastern | ≥420/≥350 | 48960/28395 | 5037/185 | 6.7 | 10.2/0.8 |
| 127 | Li et al. (2010) | Jan. 2010~May. 2010 | 20~80 | Yunnan | Southwestern | ≥420/≥350 | 818/486 | 321/72 | 30.3 | 39.0/17.2 |
| 128 | Chen et al. (2010) | Jan. 2007~Dec. 2007 | 21~92 | Yunnan | Southwestern | ≥440/≥350 | 17133/9837 | 5907/1430 | 26.7 | 34.6/14.5 |
| 129 | Fan et al. (2010) | Apr. 2009~Feb. 2010 | 20~88 | Guangxi | Southern | ≥420/≥350 | 3267/2133 | 1562/521 | 38.5 | 47.9/25.5 |
| 130 | Jiang et al. (2010) | Mar. 2009~Jun. 2009 | 20~93 | Beijing | Northern | ≥417/≥357 | 2585/1722 | 290/96 | 8.7 | 11.8/4.0 |
| 131 | Zheng et al. (2010) | Jan. 2008~Dec. 2008 | 35~85 | Zhejiang | Eastern | ≥417/≥357 | 683/837 | 71/44 | 7.2 | 10.0/5.1 |
| 132 | Hou et al. (2010) | Jan. 2007~Dec. 2007 | 18~ | Liaoning | Northeastern | ≥420/≥350 | 502/519 | 59/38 | 9.4 | 11.9/7.0 |
| 133 | Zhang et al. (2010) | Jan. 2008~Dec. 2008 | 25~ | Chongqing | Southwestern | ≥417/≥360 | 972/632 | 136/15 | 9.4 | 15.9/2.5 |
| 134 | Deng et al. (2010) | Mar. 2009~Mar. 2010 | 25~89 | Hainan | Southern | ≥417/≥357 | 21078/10570 | 4748/216 | 15.7 | 22.2/2.5 |
| 135 | Zhang et al. (2010) | Mar. 2007~Feb. 2010 | 20~58 | Xinjiang | Northwestern | ≥420/≥350 | 4264/2016 | 1764/194 | 29.7 | 38.5/9.3 |
| 136 | Yang et al. (2010) | Jan. 2007~Dec. 2007 | 60~ | Shanghai | Eastern | ≥417/≥357 | 3664/3852 | 718/747 | 16.8 | 16.6/17.1 |
| 137 | Wan et al. (2010) | Jan. 2007~Dec. 2009 | 20~ | Shanghai | Eastern | ≥416/≥357 | 2550/1013 | 681/150 | 22.4 | 25.7/13.9 |
| 138 | Zhang et al. (2010) | Jan. 2008~May. 2009 | 18~59 | Hebei | Northern | ≥417/≥357 | 2620/1180 | 444/75 | 12.3 | 15.2/5.6 |
| 139 | Wang et al. (2010) | Jan. 2008~Dec. 2008 | 35~ | Zhejiang | Eastern | ≥420/≥350 | 1510/1968 | 163/97 | 7.2 | 10.7/4.9 |
| 140 | Shen et al. (2010) | Jan. 2008~Dec. 2008 | 20~75 | Jiangsu | Eastern | ≥417/≥357 | 9240/5245 | 2052/334 | 16.6 | 21.8/8.5 |
| 141 | Chen et al. (2010) | Jan. 2009~Dec. 2009 | 20~ | Gansu | Northwestern | ≥428/≥357 | 3207/2135 | 277/29 | 5.8 | 9.0/1.5 |
| 142 | Li et al. (2010) | Jan. 2007~Dec. 2008 | 20~74 | Shanxi | Northwestern | ≥417/≥357 | 549/741 | 58/13 | 5.5 | 10.3/1.8 |
| 143 | Zhang et al. (2010) | Jan. 2008~Dec. 2008 | 15~60 | Tianjin | Northern | ≥420/≥350 | 5860/3226 | 1012/190 | 14.5 | 20.0/6.0 |
| 144 | Tan et al. (2010) | Jan. 2009~Dec. 2009 | 20~85 | Chongqing | Southwestern | ≥420/≥390 | 2529/1121 | 460/15 | 12.6 | 17.9/1.5 |
| 145 | Wang et al. (2009) | Jan. 2008~Dec. 2008 | 30~88 | Shanxi | Northern | ≥420/≥350 | 654/552 | 108/54 | 12.9 | 16.3/8.7 |
| 146 | Wu et al. (2009) | Jan. 2007~Aug. 2008 | 20~60 | Guangdong | Southern | ≥420/≥350 | 692/327 | 232/78 | 30.1 | 33.3/26.6 |
| 147 | Liu et al. (2009) | Mar. 2008~Dec. 2008 | 21~72 | Zhejiang | Eastern | ≥417/≥357 | 4877/3373 | 1487/270 | 20.3 | 30.4/7.0 |
| 148 | Huang et al. (2009) | Jan. 2008~Sept. 2008 | 21~80 | Fujian | Eastern | ≥417/≥339 | 24140/20034 | 9458/6046 | 35.2 | 38.9/32.0 |
| 149 | Jia et al. (2009) | Jan. 2007~Nov. 2007 | 20~101 | Hebei | Northern | ≥416/≥357 | 6703/1832 | 802/180 | 9.3 | 9.6/8.4 |
| 150 | Lv et al. (2009) | Feb. 2006~Dec. 2008 | 20~65 | Xinjiang | Northwestern | ≥420/≥350 | 6082/2635 | 508/51 | 6.2 | 8.1/1.8 |
| 151 | Yang et al. (2009) | Jan. 2007~May. 2009 | 24~67 | Tibet | Southwestern | ≥417/≥357 | 1874/993 | 646/136 | 26.8 | 33.7/14.0 |
| 152 | Chen et al. (2009) | Jan. 2006~Dec. 2006 | 20~ | Yunnan | Southwestern | ≥420/≥350 | 3593/3912 | 580/343 | 12.0 | 15.5/8.7 |
| 153 | Cao et al. (2009) | Jan. 2005~Dec. 2007 | 20~ | Zhejiang | Eastern | ≥417/≥357 | 9615/7639 | 2516/651 | 16.0 | 25.7/5.7 |
| 154 | Gao et al. (2008) | Jan. 2007~Dec. 2007 | 18~94 | Anhui | Eastern | ≥428/≥340 | 26066/13758 | 2883/609 | 8.6 | 11.0/3.9 |
| 155 | Chen et al. (2008) | Sept. 2006~Dec. 2007 | 18~59 | Sichuan | Southwestern | ≥417/≥357 | 1206/742 | 308/80 | 20.1 | 26.0/10.4 |
| 156 | Liu et al. (2008) | Apr. 2007~Apr. 2008 | 25~82 | Liaoning | Northeastern | ≥420/≥350 | 1144/923 | 220/139 | 13.5 | 15.5/11.5 |
| 157 | Zeng et al. (2008) | Dec. 2006~Jan. 2007 | 22~79 | Hunan | Central | ≥417/≥357 | 1346/994 | 405/103 | 19.8 | 28.3/8.8 |
| 158 | Wu et al. (2008) | Jan. 2006~Dec. 2007 | 16~ | Guangdong | Southern | ≥417/≥357 | 1366/1422 | 369/217 | 18.5 | 27.0/15.3 |
| 159 | Wang et al. (2008) | Feb. 2006~Jan. 2008 | 20~76 | Xinjiang | Northwestern | ≥417/≥357 | 2390/1824 | 502/125 | 12.7 | 18.2/4.4 |
| 160 | Wen et al. (2007) | Sept. 2006~Dec. 2006 | 35~64 | Shandong | Eastern | ≥417/≥357 | 1979/2062 | 126/44 | 4.2 | 6.4/2.0 |
| 161 | Deng et al. (2007) | Sept. 2006~Nov. 2006 | 41~93 | Liaoning | Northeastern | ≥417/≥339 | 936/218 | 251/45 | 26.3 | 28.8/17.2 |
| 162 | Li et al. (2007) | Aug. 2005~Aug. 2006 | 30~ | Jilin | Northeastern | ≥420/≥350 | 890/1485 | 147/155 | 11.5 | 15.5/9.5 |
| 163 | Miao et al. (2007) | Jan. 2000~Dec. 2000 | 20~80 | Shandong | Eastern | ≥417/≥357 | 2395/2608 | 435/225 | 12.7 | 17.9/7.9 |
| 164 | Yao et al. (2007) | Jun. 2004~Jun. 2005 | 18~85 | Shandong | Eastern | ≥417/≥357 | 2965/2693 | 273/36 | 4.8 | 8.2/1.1 |
| 165 | Huang et al. (2006) | Jan. 2000~Apr. 2006 | 20~ | Jiangsu | Eastern | ≥417/≥357 | 4950/1737 | 493/80 | 7.6 | 9.1/5.0 |
| 166 | Fang et al. (2006) | Sept. 2005~Dec. 2005 | 20~90 | Beijing | Northern | ≥417/≥357 | 1217/780 | 163/46 | 10.1 | 14.2/3.6 |
| 167 | Mao et al. (2006) | Apr. 2004~Dec. 2004 | 20~ | Zhejiang | Eastern | ≥416/≥357 | 7566/3450 | 1214/160 | 12.8 | 15.9/6.4 |
| 168 | Yu et al. (2005) | Jan. 2003~Mar. 2004 | 21~ | Guangdong | Southern | ≥417/≥357 | 7330/5994 | 1655/697 | 15.7 | 20.5/10.3 |
| 169 | Zeng et al. (2005) | Jan. 2004~Jun. 2005 | 18~85 | Guangxi | Southern | ≥417/≥357 | 2800/2400 | 490/170 | 13.9 | 19.5/7.4 |
| 170 | Yang et al. (2005) | Apr. 2000~Dec. 2000 | 18~54 | Shandong | Eastern | ≥417/≥357 | 6289/2351 | 459/78 | 5.8 | 6.9/2.8 |
| 171 | Shao et al. (2003) | Dec. 2002~Mar. 2003 | 20~ | Jiangsu | Eastern | ≥417/≥357 | 3790/3988 | 668/370 | 11.2 | 14.7/7.1 |
| 172 | Qiu et al. (2013) | Jan. 2008~Dec. 2010 | 18~ | Heilongjiang | Northeastern | ≥416/≥357 | 3725/4714 | 784/373 | 13.8 | 22.1/7.5 |
| 173 | Liu et al. (2013) | Jan. 2010~Dec. 2011 | 30~ | Tianjin | Northern | c | 5657/4793 | 1078/269 | 13.4 | 18.9/6.9 |
| 174 | Guo et al. (2015) | Oct. 2012~Sept. 2013 | 60~ | Shanghai | Eastern | ≥420/≥360 | 8541/11666 | 1465/2686 | 20.4 | 20.7/23.0 |
| 175 | Shen et al. (2018) | May. 2013~Jun. 2013 | 20~60 | Qinghai | Northwestern | ≥416/≥357 | 3014/1166 | 959/206 | 17.9 | 28.1/31.9 |
| 176 | Liu et al. (2014) | Sept.2009~Sept. 2010 | 18~ | —— | —— | ≥416/≥357 | 16019/20329 | 1585/1423 | 7.1 | 9.9/7.0 |
| 177 | Fan et al. (2019) | Nov.2015~Oct.2017 | 18~ | Huadong | Eastern | ≥420/≥350 | 62490/40693 | 6582/4613 | 10.8 | 10.5/11.3 |

**Supplementary Table 3. Summary of studies on the prevalence of hyperuricemia (Continued Supplementary Table 2)**

| **No.** | **First author (published year)** | **Response rate (%)** | **Sampling method** | **Sample source** | **Study Design** |
| --- | --- | --- | --- | --- | --- |
| 1 | Ai et al. (2019) | 100.0% | Clustered randomized | Physical examination popu. | Cross-sectional |
| 2 | Pu et al. (2019) | 100.0% | Whole sample | Occupational popu. | Cross-sectional |
| 3 | Chu et al. (2019) | 100.0% | Whole sample | Community popu. | Cross-sectional |
| 4 | Wei et al. (2019) | 100.0% | Whole sample | Community popu. | Cross-sectional |
| 5 | Tian et al. (2019) | 100.0% | Clustered randomized | Physical examination popu. | Cross-sectional |
| 6 | Zhang et al. (2019) | 100.0% | Whole sample | Occupational popu. | Cross-sectional |
| 7 | Li et al. (2018) | 100.0% | Clustered randomized | Occupational popu. | Cross-sectional |
| 8 | Zhao et al. (2018) | 100.0% | Clustered randomized | Physical examination popu. | Cross-sectional |
| 9 | Wang et al. (2018) | 100.0% | Clustered randomized | Physical examination popu. | Cross-sectional |
| 10 | Liu et al. (2018) | 100.0% | Multistage randomized | Community popu. | Cross-sectional |
| 11 | Huang et al. (2018) | 100.0% | Clustered randomized | Occupational popu. | Cross-sectional |
| 12 | Li et al. (2018) | 100.0% | Clustered randomized | Physical examination popu. | Cross-sectional |
| 13 | Zhang et al. (2018) | 100.0% | Multistage randomized | Community popu. | Cross-sectional |
| 14 | Wu et al. (2018) | 100.0% | Clustered randomized | Physical examination popu. | Cross-sectional |
| 15 | Song et al. (2018) | 100.0% | Clustered randomized | Physical examination popu. | Cross-sectional |
| 16 | Zhang et al. (2018) | 100.0% | Clustered randomized | Occupational popu. | Cross-sectional |
| 17 | Su et al. (2018) | 99.2% | Multistage randomized | Community popu. | Cross-sectional |
| 18 | Peng et al. (2018) | 100.0% | Clustered randomized | Physical examination popu. | Cross-sectional |
| 19 | Zhang et al. (2018) | 100.0% | Whole sample | Community popu. | Cross-sectional |
| 20 | Wu et al. (2017) | 91.4% | Multistage randomized | Community popu. | Cross-sectional |
| 21 | He et al. (2017) | 100.0% | Whole sample | Occupational popu. | Cross-sectional |
| 22 | Liu et al. (2017) | 72.8% | Whole sample | Occupational popu. | Cross-sectional |
| 23 | Yan et al. (2017) | 98.4% | Clustered randomized | Physical examination popu. | Cross-sectional |
| 24 | Bian et al. (2017) | 100.0% | Whole sample | Occupational popu. | Cross-sectional |
| 25 | Wang et al. (2017) | 100.0% | Clustered randomized | Physical examination popu. | Cross-sectional |
| 26 | Jiang et al. (2017) | 100.0% | Clustered randomized | Physical examination popu. | Cross-sectional |
| 27 | Kong et al. (2017) | 100.0% | Clustered randomized | Physical examination popu. | Cross-sectional |
| 28 | Feng et al. (2017) | 100.0% | Whole sample | Occupational popu. | Cross-sectional |
| 29 | Guo et al. (2017) | 100.0% | Clustered randomized | Physical examination popu. | Cross-sectional |
| 30 | Chen et al. (2017) | 94.6% | Multistage randomized | Community popu. | Cross-sectional |
| 31 | Liu et al. (2017) | 100.0% | Whole sample | Community popu. | Cross-sectional |
| 32 | Han et al. (2017) | 87.8% | Multistage randomized | Community popu. | Cross-sectional |
| 33 | Wang et al. (2017) | 84.0% | Multistage randomized | Community popu. | Cross-sectional |
| 34 | Zhang et al. (2017) | 100.0% | Clustered randomized | Occupational popu. | Cross-sectional |
| 35 | Xie et al. (2017) | 98.9% | Multistage randomized | Community popu. | Cross-sectional |
| 36 | He et al. (2017) | 100.0% | Multistage randomized | Community popu. | Cross-sectional |
| 37 | Dan et al. (2016) | 100.0% | Clustered randomized | Physical examination popu. | Cross-sectional |
| 38 | Zhang et al. (2016) | 100.0% | Clustered randomized | Physical examination popu. | Cross-sectional |
| 39 | Wang et al. (2016) | 97.9% | Multistage randomized | Community popu. | Cross-sectional |
| 40 | Wang et al. (2016) | 77.4% | Multistage randomized | Community popu. | Cross-sectional |
| 41 | Zhang et al. (2016) | 100.0% | Clustered randomized | Physical examination popu. | Cross-sectional |
| 42 | Li et al. (2016) | 100.0% | Clustered randomized | Physical examination popu. | Cross-sectional |
| 43 | Song et al. (2016) | 100.0% | Clustered randomized | Physical examination popu. | Cross-sectional |
| 44 | Xie et al. (2016) | 100.0% | Whole sample | Occupational popu. | Cross-sectional |
| 45 | Sheng et al. (2016) | 100.0% | Clustered randomized | Physical examination popu. | Cross-sectional |
| 46 | Zhang et al. (2016) | 100.0% | Multistage randomized | Occupational popu. | Cross-sectional |
| 47 | Quan et al. (2016) | 100.0% | Whole sample | Community popu. | Cross-sectional |
| 48 | Shen et al. (2016) | 100.0% | Whole sample | Occupational popu. | Cross-sectional |
| 49 | Li et al. (2016) | 100.0% | Clustered randomized | Physical examination popu. | Cross-sectional |
| 50 | Liu et al. (2015) | 100.0% | Whole sample | Occupational popu. | Cross-sectional |
| 51 | Zhou et al. (2015) | 100.0% | Clustered randomized | Occupational popu. | Cross-sectional |
| 52 | Jiang et al. (2015) | 100.0% | Clustered randomized | Physical examination popu. | Cross-sectional |
| 53 | Lv et al. (2015) | 100.0% | Clustered randomized | Occupational popu. | Cross-sectional |
| 54 | Zhang et al. (2015) | 100.0% | Clustered randomized | Physical examination popu. | Cross-sectional |
| 55 | Zhou et al. (2015) | 99.2% | Clustered randomized | Physical examination popu. | Cross-sectional |
| 56 | Fan et al. (2015) | 100.0% | Multistage randomized | Community popu. | Cross-sectional |
| 57 | Li et al. (2015) | 100.0% | Clustered randomized | Physical examination popu. | Cross-sectional |
| 58 | Lin et al. (2015) | 100.0% | Clustered randomized | Physical examination popu. | Cross-sectional |
| 59 | Zhang et al. (2015) | 100.0% | Clustered randomized | Physical examination popu. | Cross-sectional |
| 60 | Zhuo et al. (2015) | 100.0% | Clustered randomized | Physical examination popu. | Cross-sectional |
| 61 | Li et al. (2015) | 100.0% | Clustered randomized | Physical examination popu. | Cross-sectional |
| 62 | Liu et al. (2015) | 100.0% | Whole sample | Occupational popu. | Cross-sectional |
| 63 | Zhu et al. (2015) | 100.0% | Clustered randomized | Physical examination popu. | Cross-sectional |
| 64 | Mei et al. (2015) | 100.0% | Whole sample | Occupational popu. | Cross-sectional |
| 65 | Wu et al. (2015) | 95.8% | Multistage randomized | Community popu. | Cross-sectional |
| 66 | Luo et al. (2015) | 100.0% | Multistage randomized | Community popu. | Cross-sectional |
| 67 | Dong et al. (2015) | 100.0% | Whole sample | Occupational popu. | Cross-sectional |
| 68 | Han et al. (2014) | 100.0% | Clustered randomized | Physical examination popu. | Cross-sectional |
| 69 | Han et al. (2014) | 100.0% | Clustered randomized | Physical examination popu. | Cross-sectional |
| 70 | Zhang et al. (2014) | 100.0% | Clustered randomized | Physical examination popu. | Cross-sectional |
| 71 | Cui et al. (2014) | 100.0% | Clustered randomized | Physical examination popu. | Cross-sectional |
| 72 | Han et al. (2014) | 100.0% | Clustered randomized | Physical examination popu. | Cross-sectional |
| 73 | Yang et al. (2014) | 100.0% | Whole sample | Community popu. | Cross-sectional |
| 74 | Chen et al. (2014) | 100.0% | Multistage randomized | Community popu. | Cross-sectional |
| 75 | Song et al. (2014) | 94.5% | Multistage randomized | Community popu. | Cross-sectional |
| 76 | Qiang et al. (2014) | 100.0% | Multistage randomized | Physical examination popu. | Cross-sectional |
| 77 | Cao et al. (2014) | 100.0% | Whole sample | Community popu. | Cross-sectional |
| 78 | Xie et al. (2014) | 98.5% | Multistage randomized | Community popu. | Cross-sectional |
| 79 | Gao et al. (2014) | 100.0% | Clustered randomized | Physical examination popu. | Cross-sectional |
| 80 | Li et al. (2014) | 100.0% | Clustered randomized | Physical examination popu. | Cross-sectional |
| 81 | Zhang et al. (2014) | 98.6% | Multistage randomized | Community popu. | Cross-sectional |
| 82 | Liu et al. (2014) | 100.0% | Clustered randomized | Physical examination popu. | Cross-sectional |
| 83 | Huang et al. (2014) | 81.2% | Multistage randomized | Community popu. | Cross-sectional |
| 84 | Wang et al. (2013) | 100.0% | Clustered randomized | Physical examination popu. | Cross-sectional |
| 85 | Han et al. (2013) | 94.1% | Multistage randomized | Community popu. | Cross-sectional |
| 86 | Zhao et al. (2013) | 100.0% | Clustered randomized | Occupational popu. | Cross-sectional |
| 87 | Chen et al. (2013) | 74.7% | Clustered randomized | Physical examination popu. | Cross-sectional |
| 88 | Liu et al. (2013) | 100.0% | Clustered randomized | Physical examination popu. | Cross-sectional |
| 89 | Yang et al. (2013) | 100.0% | Clustered randomized | Physical examination popu. | Cross-sectional |
| 90 | Guo et al. (2013) | 100.0% | Whole sample | Occupational popu. | Cross-sectional |
| 91 | Sun et al. (2013) | 100.0% | Clustered randomized | Occupational popu. | Cross-sectional |
| 92 | Zhao et al. (2013) | 100.0% | Clustered randomized | Physical examination popu. | Cross-sectional |
| 93 | Jia et al. (2013) | 100.0% | Clustered randomized | Physical examination popu. | Cross-sectional |
| 94 | Qian et al. (2013) | 85.6% | Whole sample | Occupational popu. | Cross-sectional |
| 95 | Dong et al. (2013) | 85.3% | Multistage randomized | Community popu. | Cross-sectional |
| 96 | Wang et al. (2013) | 100.0% | Whole sample | Community popu. | Cross-sectional |
| 97 | Song et al. (2012) | 100.0% | Clustered randomized | Physical examination popu. | Cross-sectional |
| 98 | Tan et al. (2012) | 100.0% | Clustered randomized | Physical examination popu. | Cross-sectional |
| 99 | Liu et al. (2012) | 100.0% | Clustered randomized | Physical examination popu. | Cross-sectional |
| 100 | Wu et al. (2012) | 100.0% | Clustered randomized | Physical examination popu. | Cross-sectional |
| 101 | Lu et al. (2012) | 100.0% | Clustered randomized | Physical examination popu. | Cross-sectional |
| 102 | Hu et al. (2012) | 100.0% | Clustered randomized | Physical examination popu. | Cross-sectional |
| 103 | Meng et al. (2012) | 90.5% | Multistage randomized | Community popu. | Cross-sectional |
| 104 | Feng et al. (2012) | 100.0% | Whole sample | School popu. | Cross-sectional |
| 105 | Yang et al. (2012) | 100.0% | Clustered randomized | Physical examination popu. | Cross-sectional |
| 106 | Wang et al. (2012) | 100.0% | Clustered randomized | Occupational popu. | Cross-sectional |
| 107 | Wu et al. (2012) | 97.6% | Multistage randomized | Community popu. | Cross-sectional |
| 108 | She et al. (2012) | 100.0% | Clustered randomized | Physical examination popu. | Cross-sectional |
| 109 | Liu et al. (2012) | 100.0% | Clustered randomized | Physical examination popu. | Cross-sectional |
| 110 | Feng et al. (2012) | 100.0% | Clustered randomized | Physical examination popu. | Cross-sectional |
| 111 | Wang et al. (2012) | 100.0% | Clustered randomized | Community popu. | Cross-sectional |
| 112 | Qian et al. (2011) | 100.0% | Clustered randomized | Physical examination popu. | Cross-sectional |
| 113 | Wen et al. (2011) | 100.0% | Clustered randomized | Physical examination popu. | Cross-sectional |
| 114 | Huang et al. (2011) | 100.0% | Clustered randomized | Physical examination popu. | Cross-sectional |
| 115 | Liang et al. (2011) | 100.0% | Whole sample | Occupational popu. | Cross-sectional |
| 116 | Yang et al. (2011) | 100.0% | Whole sample | Occupational popu. | Cross-sectional |
| 117 | Liu et al. (2011) | 100.0% | Whole sample | Occupational popu. | Cross-sectional |
| 118 | Zhang et al. (2011) | 100.0% | Clustered randomized | Physical examination popu. | Cross-sectional |
| 119 | Huang et al. (2011) | 100.0% | Clustered randomized | Physical examination popu. | Cross-sectional |
| 120 | Chen et al. (2011) | 99.7% | Multistage randomized | Community popu. | Cross-sectional |
| 121 | Zhu et al. (2011) | 100.0% | Clustered randomized | Physical examination popu. | Cross-sectional |
| 122 | Liu et al. (2011) | 100.0% | Clustered randomized | Physical examination popu. | Cross-sectional |
| 123 | Wang et al. (2011) | 100.0% | Clustered randomized | Physical examination popu. | Cross-sectional |
| 124 | Liu et al. (2011) | 100.0% | Clustered randomized | Physical examination popu. | Cross-sectional |
| 125 | Jia et al. (2011) | 100.0% | Clustered randomized | Physical examination popu. | Cross-sectional |
| 126 | Chen et al. (2011) | 100.0% | Clustered randomized | Physical examination popu. | Cross-sectional |
| 127 | Li et al. (2010) | 100.0% | Clustered randomized | Physical examination popu. | Cross-sectional |
| 128 | Chen et al. (2010) | 100.0% | Clustered randomized | Physical examination popu. | Cross-sectional |
| 129 | Fan et al. (2010) | 100.0% | Clustered randomized | Physical examination popu. | Cross-sectional |
| 130 | Jiang et al. (2010) | 100.0% | Clustered randomized | Physical examination popu. | Cross-sectional |
| 131 | Zheng et al. (2010) | 100.0% | Multistage randomized | Community popu. | Cross-sectional |
| 132 | Hou et al. (2010) | 100.0% | Multistage randomized | Community popu. | Cross-sectional |
| 133 | Zhang et al. (2010) | 100.0% | Clustered randomized | Occupational popu. | Cross-sectional |
| 134 | Deng et al. (2010) | 100.0% | Clustered randomized | Physical examination popu. | Cross-sectional |
| 135 | Zhang et al. (2010) | 100.0% | Whole sample | Occupational popu. | Cross-sectional |
| 136 | Yang et al. (2010) | 100.0% | Whole sample | Community popu. | Cross-sectional |
| 137 | Wan et al. (2010) | 100.0% | Clustered randomized | Occupational popu. | Cross-sectional |
| 138 | Zhang et al. (2010) | 100.0% | Multistage randomized | Community popu. | Cross-sectional |
| 139 | Wang et al. (2010) | 100.0% | Multistage randomized | Community popu. | Cross-sectional |
| 140 | Shen et al. (2010) | 100.0% | Clustered randomized | Physical examination popu. | Cross-sectional |
| 141 | Chen et al. (2010) | 100.0% | Clustered randomized | Physical examination popu. | Cross-sectional |
| 142 | Li et al. (2010) | 93.4% | Multistage randomized | Community popu. | Cross-sectional |
| 143 | Zhang et al. (2010) | 100.0% | Clustered randomized | Physical examination popu. | Cross-sectional |
| 144 | Tan et al. (2010) | 100.0% | Clustered randomized | Physical examination popu. | Cross-sectional |
| 145 | Wang et al. (2009) | 100.0% | Clustered randomized | Physical examination popu. | Cross-sectional |
| 146 | Wu et al. (2009) | 100.0% | Clustered randomized | Physical examination popu. | Cross-sectional |
| 147 | Liu et al. (2009) | 100.0% | Clustered randomized | Occupational popu. | Cross-sectional |
| 148 | Huang et al. (2009) | 100.0% | Clustered randomized | Physical examination popu. | Cross-sectional |
| 149 | Jia et al. (2009) | 100.0% | Whole sample | Occupational popu. | Cross-sectional |
| 150 | Lv et al. (2009) | 100.0% | Clustered randomized | Physical examination popu. | Cross-sectional |
| 151 | Yang et al. (2009) | 100.0% | Clustered randomized | Physical examination popu. | Cross-sectional |
| 152 | Chen et al. (2009) | 100.0% | Multistage randomized | Community popu. | Cross-sectional |
| 153 | Cao et al. (2009) | 100.0% | Whole sample | Occupational popu. | Cross-sectional |
| 154 | Gao et al. (2008) | 100.0% | Multistage randomized | Community popu. | Cross-sectional |
| 155 | Chen et al. (2008) | 100.0% | Clustered randomized | Occupational popu. | Cross-sectional |
| 156 | Liu et al. (2008) | 100.0% | Clustered randomized | Physical examination popu. | Cross-sectional |
| 157 | Zeng et al. (2008) | 80.3% | Multistage randomized | Occupational popu. | Cross-sectional |
| 158 | Wu et al. (2008) | 100.0% | Multistage randomized | Community popu. | Cross-sectional |
| 159 | Wang et al. (2008) | 100.0% | Clustered randomized | Physical examination popu. | Cross-sectional |
| 160 | Wen et al. (2007) | 95.7% | Multistage randomized | Community popu. | Cross-sectional |
| 161 | Deng et al. (2007) | 100.0% | Clustered randomized | Physical examination popu. | Cross-sectional |
| 162 | Li et al. (2007) | 95.4% | Multistage randomized | Community popu. | Cross-sectional |
| 163 | Miao et al. (2007) | 91.0% | Multistage randomized | Community popu. | Cross-sectional |
| 164 | Yao et al. (2007) | 96.3% | Whole sample | Community popu. | Cross-sectional |
| 165 | Huang et al. (2006) | 100.0% | Clustered randomized | Physical examination popu. | Cross-sectional |
| 166 | Fang et al. (2006) | 100.0% | Clustered randomized | Occupational popu. | Cross-sectional |
| 167 | Mao et al. (2006) | 98.5% | Whole sample | Occupational popu. | Cross-sectional |
| 168 | Yu et al. (2005) | 100.0% | Clustered randomized | Physical examination popu. | Cross-sectional |
| 169 | Zeng et al. (2005) | 100.0% | Whole sample | Occupational popu. | Cross-sectional |
| 170 | Yang et al. (2005) | 100.0% | Multistage randomized | Occupational popu. | Cross-sectional |
| 171 | Shao et al. (2003) | 96.1% | Multistage randomized | Community popu. | Cross-sectional |
| 172 | Qiu et al. (2013) | 100.0% | Multistage randomized | Community popu. | Cross-sectional |
| 173 | Liu et al. (2013) | 100.0% | Multistage randomized | Community popu. | Cross-sectional |
| 174 | Guo et al. (2015) | 100.0% | Clustered randomized | Community popu. | Cross-sectional |
| 175 | Shen et al. (2018) | 84.3% | Multistage randomized | Occupational popu. | Cross-sectional |
| 176 | Liu et al. (2014) | 71.9% | Multistage randomized | Community popu. | Cross-sectional |
| 177 | Fan et al. (2019) | 100.0% | Clustered randomized | Physical examination popu. | Cross-sectional |

**Supplementary Table 4. Quality appraisal of 177 eligible studies**

| **No.** | **First author (published year)** | **Quality appraisal** | | | | | | | | | | | **Total score** |
| --- | --- | --- | --- | --- | --- | --- | --- | --- | --- | --- | --- | --- | --- |
|  |  | **Item 1** | **Item 2** | **Item 3** | **Item 4** | **Item 5** | **Item 6** | **Item 7** | **Item 8** | **Item 9** | **Item 10** | **Item 11** |  |
| 1 | Ai et al. (2019) | 1 | 1 | 1 | 0 | 1 | 0 | 0 | 1 | 1 | 1 | 0 | 7 |
| 2 | Pu et al. (2019) | 1 | 1 | 1 | 0 | 1 | 1 | 0 | 0 | 1 | 1 | 0 | 7 |
| 3 | Chu et al. (2019) | 1 | 1 | 1 | 1 | 1 | 1 | 0 | 1 | 0 | 1 | 0 | 8 |
| 4 | Wei et al. (2019) | 1 | 1 | 1 | 1 | 1 | 1 | 1 | 1 | 0 | 1 | 0 | 9 |
| 5 | Tian et al. (2019) | 1 | 1 | 1 | 0 | 1 | 0 | 0 | 1 | 1 | 1 | 0 | 7 |
| 6 | Zhang et al. (2019) | 1 | 1 | 1 | 0 | 1 | 1 | 0 | 0 | 1 | 1 | 0 | 7 |
| 7 | Li et al. (2018) | 1 | 1 | 1 | 0 | 1 | 0 | 0 | 1 | 0 | 1 | 0 | 6 |
| 8 | Zhao et al. (2018) | 1 | 1 | 1 | 0 | 1 | 0 | 0 | 0 | 1 | 1 | 0 | 6 |
| 9 | Wang et al. (2018) | 1 | 1 | 1 | 0 | 1 | 0 | 1 | 1 | 1 | 1 | 0 | 8 |
| 10 | Liu et al. (2018) | 1 | 1 | 1 | 1 | 1 | 1 | 0 | 1 | 1 | 1 | 0 | 9 |
| 11 | Huang et al. (2018) | 1 | 1 | 1 | 0 | 1 | 1 | 1 | 1 | 0 | 1 | 0 | 8 |
| 12 | Li et al. (2018) | 1 | 1 | 1 | 0 | 1 | 0 | 1 | 1 | 1 | 1 | 0 | 8 |
| 13 | Zhang et al. (2018) | 1 | 1 | 1 | 1 | 1 | 1 | 1 | 1 | 1 | 1 | 0 | 10 |
| 14 | Wu et al. (2018) | 1 | 1 | 1 | 0 | 1 | 1 | 0 | 0 | 1 | 1 | 0 | 7 |
| 15 | Song et al. (2018) | 1 | 1 | 1 | 0 | 1 | 0 | 0 | 1 | 1 | 1 | 0 | 7 |
| 16 | Zhang et al. (2018) | 1 | 1 | 1 | 0 | 1 | 0 | 1 | 1 | 1 | 1 | 0 | 8 |
| 17 | Su et al. (2018) | 1 | 1 | 1 | 1 | 1 | 1 | 1 | 1 | 0 | 1 | 0 | 9 |
| 18 | Peng et al. (2018) | 1 | 1 | 1 | 0 | 1 | 1 | 0 | 0 | 1 | 1 | 0 | 7 |
| 19 | Zhang et al. (2018) | 1 | 1 | 1 | 1 | 1 | 0 | 1 | 1 | 0 | 1 | 0 | 8 |
| 20 | Wu et al. (2017) | 1 | 1 | 1 | 1 | 1 | 1 | 1 | 1 | 0 | 1 | 0 | 9 |
| 21 | He et al. (2017) | 1 | 1 | 1 | 0 | 1 | 1 | 1 | 1 | 0 | 1 | 0 | 8 |
| 22 | Liu et al. (2017) | 1 | 1 | 1 | 0 | 1 | 1 | 0 | 1 | 0 | 1 | 0 | 7 |
| 23 | Yan et al. (2017) | 1 | 1 | 1 | 0 | 1 | 0 | 0 | 1 | 1 | 1 | 0 | 7 |
| 24 | Bian et al. (2017) | 1 | 1 | 1 | 0 | 1 | 1 | 1 | 0 | 1 | 1 | 0 | 8 |
| 25 | Wang et al. (2017) | 1 | 1 | 1 | 0 | 1 | 0 | 0 | 1 | 0 | 1 | 0 | 6 |
| 26 | Jiang et al. (2017) | 1 | 1 | 1 | 0 | 1 | 0 | 1 | 1 | 0 | 1 | 0 | 7 |
| 27 | Kong et al. (2017) | 1 | 1 | 1 | 0 | 1 | 1 | 0 | 0 | 1 | 1 | 0 | 7 |
| 28 | Feng et al. (2017) | 1 | 1 | 1 | 0 | 1 | 1 | 0 | 1 | 0 | 1 | 0 | 7 |
| 29 | Guo et al. (2017) | 1 | 1 | 1 | 0 | 1 | 0 | 0 | 0 | 1 | 1 | 0 | 6 |
| 30 | Chen et al. (2017) | 1 | 1 | 1 | 1 | 1 | 1 | 0 | 1 | 0 | 1 | 0 | 8 |
| 31 | Liu et al. (2017) | 1 | 1 | 1 | 1 | 1 | 1 | 0 | 1 | 1 | 1 | 0 | 9 |
| 32 | Han et al. (2017) | 1 | 1 | 1 | 1 | 1 | 1 | 1 | 1 | 1 | 1 | 0 | 10 |
| 33 | Wang et al. (2017) | 1 | 1 | 1 | 1 | 1 | 1 | 0 | 1 | 1 | 1 | 0 | 9 |
| 34 | Zhang et al. (2017) | 1 | 1 | 1 | 0 | 1 | 0 | 0 | 1 | 1 | 1 | 0 | 7 |
| 35 | Xie et al. (2017) | 1 | 1 | 1 | 1 | 1 | 1 | 1 | 1 | 0 | 1 | 0 | 9 |
| 36 | He et al. (2017) | 1 | 1 | 1 | 1 | 1 | 1 | 0 | 1 | 0 | 1 | 0 | 8 |
| 37 | Dan et al. (2016) | 1 | 1 | 1 | 0 | 1 | 0 | 0 | 0 | 1 | 1 | 0 | 6 |
| 38 | Zhang et al. (2016) | 1 | 1 | 1 | 0 | 1 | 1 | 0 | 0 | 1 | 1 | 0 | 7 |
| 39 | Wang et al. (2016) | 1 | 1 | 1 | 1 | 1 | 1 | 1 | 1 | 0 | 1 | 0 | 9 |
| 40 | Wang et al. (2016) | 1 | 1 | 1 | 1 | 1 | 1 | 1 | 1 | 0 | 1 | 0 | 9 |
| 41 | Zhang et al. (2016) | 1 | 1 | 1 | 0 | 1 | 0 | 1 | 1 | 1 | 1 | 0 | 8 |
| 42 | Li et al. (2016) | 1 | 1 | 1 | 0 | 1 | 0 | 0 | 1 | 0 | 1 | 0 | 6 |
| 43 | Song et al. (2016) | 1 | 1 | 1 | 0 | 1 | 0 | 1 | 1 | 1 | 1 | 0 | 8 |
| 44 | Xie et al. (2016) | 1 | 1 | 1 | 0 | 1 | 1 | 0 | 1 | 1 | 1 | 0 | 8 |
| 45 | Sheng et al. (2016) | 1 | 1 | 1 | 0 | 1 | 0 | 0 | 0 | 1 | 1 | 0 | 6 |
| 46 | Zhang et al. (2016) | 1 | 1 | 1 | 0 | 1 | 1 | 0 | 1 | 1 | 1 | 0 | 8 |
| 47 | Quan et al. (2016) | 1 | 1 | 1 | 1 | 1 | 1 | 0 | 1 | 0 | 1 | 0 | 8 |
| 48 | Shen et al. (2016) | 1 | 1 | 1 | 0 | 1 | 1 | 1 | 1 | 0 | 1 | 0 | 8 |
| 49 | Li et al. (2016) | 1 | 1 | 1 | 0 | 1 | 1 | 0 | 0 | 1 | 1 | 0 | 7 |
| 50 | Liu et al. (2015) | 1 | 1 | 1 | 0 | 1 | 1 | 0 | 1 | 0 | 1 | 0 | 7 |
| 51 | Zhou et al. (2015) | 1 | 1 | 1 | 0 | 1 | 1 | 0 | 0 | 1 | 1 | 0 | 7 |
| 52 | Jiang et al. (2015) | 1 | 1 | 1 | 0 | 1 | 1 | 0 | 0 | 1 | 1 | 0 | 7 |
| 53 | Lv et al. (2015) | 1 | 1 | 1 | 0 | 1 | 1 | 0 | 0 | 1 | 1 | 0 | 7 |
| 54 | Zhang et al. (2015) | 1 | 1 | 1 | 0 | 1 | 1 | 1 | 0 | 1 | 1 | 0 | 8 |
| 55 | Zhou et al. (2015) | 1 | 1 | 1 | 0 | 1 | 1 | 0 | 0 | 1 | 1 | 0 | 7 |
| 56 | Fan et al. (2015) | 1 | 1 | 1 | 1 | 1 | 1 | 1 | 1 | 0 | 1 | 0 | 9 |
| 57 | Li et al. (2015) | 1 | 1 | 1 | 0 | 1 | 0 | 0 | 1 | 1 | 1 | 0 | 7 |
| 58 | Lin et al. (2015) | 1 | 1 | 1 | 0 | 1 | 0 | 1 | 1 | 1 | 1 | 0 | 8 |
| 59 | Zhang et al. (2015) | 1 | 1 | 1 | 0 | 1 | 1 | 0 | 0 | 1 | 1 | 0 | 7 |
| 60 | Zhuo et al. (2015) | 1 | 1 | 1 | 0 | 1 | 1 | 1 | 1 | 0 | 1 | 0 | 8 |
| 61 | Li et al. (2015) | 1 | 1 | 1 | 0 | 1 | 1 | 1 | 1 | 0 | 1 | 0 | 8 |
| 62 | Liu et al. (2015) | 1 | 1 | 1 | 0 | 1 | 1 | 1 | 0 | 1 | 1 | 0 | 8 |
| 63 | Zhu et al. (2015) | 1 | 1 | 1 | 0 | 1 | 1 | 1 | 0 | 1 | 1 | 0 | 8 |
| 64 | Mei et al. (2015) | 1 | 1 | 1 | 0 | 1 | 1 | 0 | 1 | 1 | 1 | 0 | 8 |
| 65 | Wu et al. (2015) | 1 | 1 | 1 | 1 | 1 | 1 | 1 | 1 | 0 | 1 | 0 | 9 |
| 66 | Luo et al. (2015) | 1 | 1 | 1 | 1 | 1 | 1 | 1 | 1 | 1 | 1 | 0 | 10 |
| 67 | Dong et al. (2015) | 1 | 1 | 1 | 0 | 1 | 1 | 0 | 0 | 1 | 1 | 0 | 7 |
| 68 | Han et al. (2014) | 1 | 1 | 1 | 0 | 1 | 0 | 1 | 1 | 1 | 1 | 0 | 8 |
| 69 | Han et al. (2014) | 1 | 1 | 1 | 0 | 1 | 0 | 1 | 1 | 1 | 1 | 0 | 8 |
| 70 | Zhang et al. (2014) | 1 | 1 | 1 | 0 | 1 | 0 | 1 | 1 | 1 | 1 | 0 | 8 |
| 71 | Cui et al. (2014) | 1 | 1 | 1 | 0 | 1 | 0 | 1 | 1 | 1 | 1 | 0 | 8 |
| 72 | Han et al. (2014) | 1 | 1 | 1 | 0 | 1 | 0 | 0 | 1 | 1 | 1 | 0 | 7 |
| 73 | Yang et al. (2014) | 1 | 1 | 1 | 1 | 1 | 0 | 1 | 0 | 1 | 1 | 0 | 8 |
| 74 | Chen et al. (2014) | 1 | 1 | 1 | 1 | 1 | 1 | 1 | 1 | 0 | 1 | 0 | 9 |
| 75 | Song et al. (2014) | 1 | 1 | 1 | 1 | 1 | 1 | 1 | 1 | 0 | 1 | 0 | 9 |
| 76 | Qiang et al. (2014) | 1 | 1 | 1 | 0 | 1 | 1 | 1 | 1 | 1 | 1 | 0 | 9 |
| 77 | Cao et al. (2014) | 1 | 1 | 1 | 1 | 1 | 1 | 1 | 1 | 1 | 1 | 0 | 10 |
| 78 | Xie et al. (2014) | 1 | 1 | 1 | 1 | 1 | 1 | 1 | 1 | 1 | 1 | 0 | 10 |
| 79 | Gao et al. (2014) | 1 | 1 | 1 | 0 | 1 | 0 | 0 | 1 | 1 | 1 | 0 | 7 |
| 80 | Li et al. (2014) | 1 | 1 | 1 | 0 | 1 | 1 | 0 | 1 | 0 | 1 | 0 | 7 |
| 81 | Zhang et al. (2014) | 1 | 1 | 1 | 1 | 1 | 1 | 1 | 1 | 0 | 1 | 0 | 9 |
| 82 | Liu et al. (2014) | 1 | 1 | 1 | 0 | 1 | 0 | 1 | 1 | 1 | 1 | 0 | 8 |
| 83 | Huang et al. (2014) | 1 | 1 | 1 | 1 | 1 | 1 | 1 | 1 | 1 | 1 | 0 | 10 |
| 84 | Wang et al. (2013) | 1 | 1 | 1 | 0 | 1 | 0 | 1 | 0 | 1 | 1 | 0 | 7 |
| 85 | Han et al. (2013) | 1 | 1 | 1 | 1 | 1 | 0 | 1 | 1 | 1 | 1 | 0 | 9 |
| 86 | Zhao et al. (2013) | 1 | 1 | 1 | 0 | 1 | 1 | 1 | 0 | 1 | 1 | 0 | 8 |
| 87 | Chen et al. (2013) | 1 | 1 | 1 | 0 | 1 | 0 | 1 | 1 | 0 | 1 | 0 | 7 |
| 88 | Liu et al. (2013) | 1 | 1 | 1 | 0 | 1 | 1 | 0 | 0 | 1 | 1 | 0 | 7 |
| 89 | Yang et al. (2013) | 1 | 1 | 1 | 0 | 1 | 0 | 0 | 1 | 1 | 1 | 0 | 7 |
| 90 | Guo et al. (2013) | 1 | 1 | 1 | 0 | 1 | 1 | 1 | 0 | 1 | 1 | 0 | 8 |
| 91 | Sun et al. (2013) | 1 | 1 | 1 | 0 | 1 | 0 | 1 | 0 | 1 | 1 | 0 | 7 |
| 92 | Zhao et al. (2013) | 1 | 1 | 1 | 0 | 1 | 0 | 1 | 1 | 1 | 1 | 0 | 8 |
| 93 | Jia et al. (2013) | 1 | 1 | 1 | 0 | 1 | 0 | 1 | 1 | 1 | 1 | 0 | 8 |
| 94 | Qian et al. (2013) | 1 | 1 | 1 | 1 | 1 | 1 | 1 | 1 | 0 | 1 | 0 | 9 |
| 95 | Dong et al. (2013) | 1 | 1 | 1 | 1 | 1 | 1 | 1 | 1 | 0 | 1 | 0 | 9 |
| 96 | Wang et al. (2013) | 1 | 1 | 1 | 1 | 1 | 1 | 0 | 1 | 0 | 1 | 0 | 8 |
| 97 | Song et al. (2012) | 1 | 1 | 1 | 0 | 1 | 0 | 0 | 0 | 1 | 1 | 0 | 6 |
| 98 | Tan et al. (2012) | 1 | 1 | 1 | 0 | 1 | 0 | 0 | 1 | 0 | 1 | 0 | 6 |
| 99 | Liu et al. (2012) | 1 | 1 | 1 | 0 | 1 | 1 | 1 | 1 | 0 | 1 | 0 | 8 |
| 100 | Wu et al. (2012) | 1 | 1 | 1 | 0 | 1 | 0 | 0 | 1 | 1 | 1 | 0 | 7 |
| 101 | Lu et al. (2012) | 1 | 1 | 1 | 0 | 1 | 0 | 0 | 1 | 0 | 1 | 0 | 6 |
| 102 | Hu et al. (2012) | 1 | 1 | 1 | 0 | 1 | 1 | 0 | 0 | 1 | 1 | 0 | 7 |
| 103 | Meng et al. (2012) | 1 | 1 | 1 | 1 | 1 | 1 | 1 | 0 | 1 | 1 | 0 | 9 |
| 104 | Feng et al. (2012) | 1 | 1 | 1 | 0 | 1 | 1 | 0 | 0 | 1 | 1 | 0 | 7 |
| 105 | Yang et al. (2012) | 1 | 1 | 1 | 0 | 1 | 0 | 1 | 1 | 0 | 1 | 0 | 7 |
| 106 | Wang et al. (2012) | 1 | 1 | 1 | 0 | 1 | 0 | 1 | 0 | 1 | 1 | 0 | 7 |
| 107 | Wu et al. (2012) | 1 | 1 | 1 | 1 | 1 | 0 | 1 | 1 | 1 | 1 | 0 | 9 |
| 108 | She et al. (2012) | 1 | 1 | 1 | 0 | 1 | 1 | 1 | 1 | 0 | 1 | 0 | 8 |
| 109 | Liu et al. (2012) | 1 | 1 | 1 | 0 | 1 | 0 | 1 | 1 | 0 | 1 | 0 | 7 |
| 110 | Feng et al. (2012) | 1 | 1 | 1 | 0 | 1 | 0 | 0 | 0 | 1 | 1 | 0 | 6 |
| 111 | Wang et al. (2012) | 1 | 1 | 1 | 1 | 1 | 0 | 0 | 0 | 1 | 1 | 0 | 7 |
| 112 | Qian et al. (2011) | 1 | 1 | 1 | 0 | 1 | 0 | 0 | 0 | 1 | 1 | 0 | 6 |
| 113 | Wen et al. (2011) | 1 | 1 | 1 | 0 | 1 | 1 | 0 | 0 | 1 | 1 | 0 | 7 |
| 114 | Huang et al. (2011) | 1 | 1 | 1 | 0 | 1 | 0 | 0 | 1 | 1 | 1 | 0 | 7 |
| 115 | Liang et al. (2011) | 1 | 1 | 1 | 0 | 1 | 1 | 0 | 1 | 0 | 1 | 0 | 7 |
| 116 | Yang et al. (2011) | 1 | 1 | 1 | 0 | 1 | 1 | 0 | 1 | 0 | 1 | 0 | 7 |
| 117 | Liu et al. (2011) | 1 | 1 | 1 | 0 | 1 | 1 | 0 | 1 | 1 | 1 | 0 | 8 |
| 118 | Zhang et al. (2011) | 1 | 1 | 1 | 0 | 1 | 0 | 0 | 1 | 0 | 1 | 0 | 6 |
| 119 | Huang et al. (2011) | 1 | 1 | 1 | 0 | 1 | 0 | 1 | 1 | 1 | 1 | 0 | 8 |
| 120 | Chen et al. (2011) | 1 | 1 | 1 | 1 | 1 | 0 | 1 | 1 | 1 | 1 | 0 | 9 |
| 121 | Zhu et al. (2011) | 1 | 1 | 1 | 0 | 1 | 1 | 0 | 0 | 1 | 1 | 0 | 7 |
| 122 | Liu et al. (2011) | 1 | 1 | 1 | 0 | 1 | 0 | 1 | 1 | 1 | 1 | 0 | 8 |
| 123 | Wang et al. (2011) | 1 | 1 | 1 | 0 | 1 | 1 | 1 | 1 | 0 | 1 | 0 | 8 |
| 124 | Liu et al. (2011) | 1 | 1 | 1 | 0 | 1 | 1 | 1 | 0 | 1 | 1 | 0 | 8 |
| 125 | Jia et al. (2011) | 1 | 1 | 1 | 0 | 1 | 1 | 1 | 1 | 0 | 1 | 0 | 8 |
| 126 | Chen et al. (2011) | 1 | 1 | 1 | 0 | 1 | 0 | 1 | 1 | 1 | 1 | 0 | 8 |
| 127 | Li et al. (2010) | 1 | 1 | 1 | 0 | 1 | 0 | 0 | 0 | 1 | 1 | 0 | 6 |
| 128 | Chen et al. (2010) | 1 | 1 | 1 | 0 | 1 | 1 | 1 | 1 | 0 | 1 | 0 | 8 |
| 129 | Fan et al. (2010) | 1 | 1 | 1 | 0 | 1 | 1 | 1 | 1 | 0 | 1 | 0 | 8 |
| 130 | Jiang et al. (2010) | 1 | 1 | 1 | 0 | 1 | 0 | 1 | 0 | 1 | 1 | 0 | 7 |
| 131 | Zheng et al. (2010) | 1 | 1 | 1 | 1 | 1 | 1 | 0 | 1 | 0 | 1 | 0 | 8 |
| 132 | Hou et al. (2010) | 1 | 1 | 1 | 1 | 1 | 1 | 1 | 1 | 0 | 1 | 0 | 9 |
| 133 | Zhang et al. (2010) | 1 | 1 | 1 | 0 | 1 | 0 | 1 | 0 | 1 | 1 | 0 | 7 |
| 134 | Deng et al. (2010) | 1 | 1 | 1 | 0 | 1 | 0 | 1 | 1 | 1 | 1 | 0 | 8 |
| 135 | Zhang et al. (2010) | 1 | 1 | 1 | 0 | 1 | 1 | 0 | 1 | 1 | 1 | 0 | 8 |
| 136 | Yang et al. (2010) | 1 | 1 | 1 | 1 | 1 | 1 | 1 | 1 | 0 | 1 | 0 | 9 |
| 137 | Wan et al. (2010) | 1 | 1 | 1 | 0 | 1 | 0 | 0 | 1 | 0 | 1 | 0 | 6 |
| 138 | Zhang et al. (2010) | 1 | 1 | 1 | 1 | 1 | 0 | 1 | 1 | 1 | 1 | 0 | 9 |
| 139 | Wang et al. (2010) | 1 | 1 | 1 | 1 | 1 | 1 | 1 | 1 | 0 | 1 | 0 | 9 |
| 140 | Shen et al. (2010) | 1 | 1 | 1 | 0 | 1 | 0 | 0 | 1 | 1 | 1 | 0 | 7 |
| 141 | Chen et al. (2010) | 1 | 1 | 1 | 0 | 1 | 0 | 1 | 1 | 1 | 1 | 0 | 8 |
| 142 | Li et al. (2010) | 1 | 1 | 1 | 1 | 1 | 1 | 1 | 1 | 0 | 1 | 0 | 9 |
| 143 | Zhang et al. (2010) | 1 | 1 | 1 | 0 | 1 | 1 | 1 | 1 | 0 | 1 | 0 | 8 |
| 144 | Tan et al. (2010) | 1 | 1 | 1 | 0 | 1 | 0 | 1 | 0 | 1 | 1 | 0 | 7 |
| 145 | Wang et al. (2009) | 1 | 1 | 1 | 0 | 1 | 0 | 0 | 0 | 1 | 1 | 0 | 6 |
| 146 | Wu et al. (2009) | 1 | 1 | 1 | 0 | 1 | 0 | 1 | 1 | 0 | 1 | 0 | 7 |
| 147 | Liu et al. (2009) | 1 | 1 | 1 | 0 | 1 | 0 | 0 | 1 | 1 | 1 | 0 | 7 |
| 148 | Huang et al. (2009) | 1 | 1 | 1 | 0 | 1 | 1 | 0 | 0 | 1 | 1 | 0 | 7 |
| 149 | Jia et al. (2009) | 1 | 1 | 1 | 0 | 1 | 1 | 1 | 0 | 1 | 1 | 0 | 8 |
| 150 | Lv et al. (2009) | 1 | 1 | 1 | 0 | 1 | 1 | 0 | 0 | 1 | 1 | 0 | 7 |
| 151 | Yang et al. (2009) | 1 | 1 | 1 | 0 | 1 | 0 | 1 | 0 | 1 | 1 | 0 | 7 |
| 152 | Chen et al. (2009) | 1 | 1 | 1 | 1 | 1 | 1 | 0 | 1 | 1 | 1 | 0 | 9 |
| 153 | Cao et al. (2009) | 1 | 1 | 1 | 0 | 1 | 1 | 1 | 1 | 1 | 1 | 0 | 9 |
| 154 | Gao et al. (2008) | 1 | 1 | 1 | 1 | 1 | 1 | 1 | 1 | 1 | 1 | 0 | 10 |
| 155 | Chen et al. (2008) | 1 | 1 | 1 | 0 | 1 | 0 | 1 | 1 | 0 | 1 | 0 | 7 |
| 156 | Liu et al. (2008) | 1 | 1 | 1 | 0 | 1 | 0 | 1 | 0 | 1 | 1 | 0 | 7 |
| 157 | Zeng et al. (2008) | 1 | 1 | 1 | 0 | 1 | 1 | 1 | 1 | 0 | 1 | 0 | 8 |
| 158 | Wu et al. (2008) | 1 | 1 | 1 | 1 | 1 | 1 | 1 | 1 | 0 | 1 | 0 | 9 |
| 159 | Wang et al. (2008) | 1 | 1 | 1 | 0 | 1 | 0 | 0 | 0 | 1 | 1 | 0 | 6 |
| 160 | Wen et al. (2007) | 1 | 1 | 1 | 1 | 1 | 1 | 1 | 1 | 0 | 1 | 0 | 9 |
| 161 | Deng et al. (2007) | 1 | 1 | 1 | 0 | 1 | 0 | 1 | 0 | 1 | 1 | 0 | 7 |
| 162 | Li et al. (2007) | 1 | 1 | 1 | 1 | 1 | 1 | 1 | 1 | 0 | 1 | 0 | 9 |
| 163 | Miao et al. (2007) | 1 | 1 | 1 | 1 | 1 | 1 | 1 | 1 | 1 | 1 | 0 | 10 |
| 164 | Yao et al. (2007) | 1 | 1 | 1 | 1 | 1 | 1 | 1 | 1 | 1 | 1 | 0 | 10 |
| 165 | Huang et al. (2006) | 1 | 1 | 1 | 0 | 1 | 0 | 0 | 1 | 1 | 1 | 0 | 7 |
| 166 | Fang et al. (2006) | 1 | 1 | 1 | 0 | 1 | 0 | 1 | 1 | 0 | 1 | 0 | 7 |
| 167 | Mao et al. (2006) | 1 | 1 | 1 | 0 | 1 | 1 | 1 | 1 | 1 | 1 | 0 | 9 |
| 168 | Yu et al. (2005) | 1 | 1 | 1 | 0 | 1 | 0 | 1 | 1 | 1 | 1 | 0 | 8 |
| 169 | Zeng et al. (2005) | 1 | 1 | 1 | 0 | 1 | 1 | 0 | 1 | 1 | 1 | 0 | 8 |
| 170 | Yang et al. (2005) | 1 | 1 | 1 | 0 | 1 | 1 | 0 | 1 | 1 | 1 | 0 | 8 |
| 171 | Shao et al. (2003) | 1 | 1 | 1 | 1 | 1 | 1 | 1 | 1 | 1 | 1 | 0 | 10 |
| 172 | Qiu et al. (2013) | 1 | 1 | 1 | 1 | 1 | 1 | 1 | 1 | 1 | 1 | 0 | 10 |
| 173 | Liu et al. (2013) | 1 | 1 | 1 | 1 | 1 | 1 | 1 | 1 | 1 | 1 | 0 | 10 |
| 174 | Guo et al. (2015) | 1 | 1 | 1 | 1 | 1 | 1 | 1 | 1 | 0 | 1 | 0 | 9 |
| 175 | Shen et al. (2018) | 1 | 1 | 1 | 0 | 1 | 1 | 1 | 1 | 0 | 1 | 0 | 8 |
| 176 | Liu et al. (2014) | 1 | 1 | 1 | 1 | 1 | 1 | 1 | 0 | 1 | 1 | 0 | 9 |
| 177 | Fan et al. (2019) | 1 | 1 | 1 | 0 | 1 | 0 | 1 | 1 | 1 | 1 | 0 | 8 |

**Supplementary Text 1. Reference of 177 eligible studies**

[1] AI Q, YUAN J, ZHANG F, et al. Analysis of serum uric acid level and related factors in 5776 healthy subjects (Chinese). Inner Mongolia Medical Journal. 2019;51(03):268-269.

[2] PU W. Clinical investigation on the hyperuricemia incidence in colleges (Chinese). Public Medical Forum Magazine. 2019 (23):3302-3304.

[3] CHU A, ZHENG L, CEN H, et al. Prevalence and related factor analysis on hyperuricemia in elderly in Jinshan District, Shanghai (Chinese). Shanghai Journal of Preventive Medicine. 2019;31(03):183-187.

[4] WEI R, ZHANG Y, ZHANG F, et al. Prevalence and associated factors of hyperuricemia and gout in elderly people in Xujiahui subdistrict, Shanghai (Chinese). Chinese General Practice. 2019;22(16):1954-1959+1972.

[5] TIAN Q, WANG Y, LI Z, et al. Correlation between uric acid and risk factors of cardiovascular disease in people under physical examination (Chinese). China Continuing Medical Education. 2019;11(1):62-65.

[6] ZHANG H. Analysis of hyperuricemia among bank employees in Guiyang (Chinese). Special Health. 2019 (13):29.

[7] LI X, HAN Y, YU D, et al. Analysis of 3144 civil servants over 45 years old on high uric acid, hypertension and hyperlipidemia (Chinese). Ningxia Medical Journal. 2018;40(12):1215-1217.

[8] ZHAO D, JIN Q, HU E, et al. Current condition of serum uric acid concentration in health examination population of Bengbu (Chinese). Journal of Huaihai Medicine. 2018;36(01):13-15.

[9] WANG D, YIN Y, YU S, et al. Survey for variation of prevalence of hyperuricemia and its correlations with blood glucose and lipid in healthy adults receiving physical examination at Peking Union Medical College Hospital from 2012 to 2017 (Chinese). Chinese Journal of Clinical Laboratory Science. 2018;36(06):462-466.

[10] LIU Y, ZHAO G, CHEN Q, et al. Analysis of serum uric acid level and related factors in 10077 Zhuang people in Guangxi (Chinese). Diabetes World. 2018;15(3):6-7.

[11] HUANG L, YANG Y, ZENG H, et al. Associated influencing factors of hyperuricemia in some civil servants in Guangzhou city (Chinese). Journal of Guizhou Medical University. 2018;43(08):949-953.

[12] LI S, CHEN Y, DONG L, et al. Epidemiological status and influencing factors of hyperuricemia in Zhangjiakou of Hebei province (Chinese). Journal of Medical Pest Control. 2018;34(05):455-457.

[13] ZHANG H. Prevalence of hyperuricemia and its influence factors in rural area of Henan (Chinese) [Master]: Zhengzhou University; 2018.

[14] WU J, PENG Z, CAO X, et al. Effect of serum uric acid level on blood routine parameters in healthy population (Chinese). Hainan Medical Journal. 2018;29(17):2428-2431.

[15] SONG J, SUN Y, HUANG L, et al. Prevalence of hyperuricemia and the correlation between uric acid and blood lipid (Chinese) Laboratory Medicine and Clinic. 2018;15(12):1841-1843.

[16] ZHANG H, ZHAO Q, MENG L, et al. Relationship between hyperuricemia and metabolic syndrome in ningxia health examination population (Chinese). Chinese Journal of Prevention and Control of Chronic Diseases. 2018;26(09):660-663.

[17] SU Y, NI J, ZHENG J, et al. Investigation of metabolic syndrome and related chronic diseases in Ouhai residents (Chinese). Chinese Journal of Prevention and Control of Chronic Diseases. 2018;26(8):597-600.

[18] PENG S, WANG H, LIU Q, et al. Analysis of uric acid level in physical examination population in Pudong area of Shanghai (Chinese). The Journal of Medical Theory and Practice. 2018;31(20):3139-3140.

[19] ZHANG Y. Study on risk factors of hyperuricemia in the people in shuimogou area of Ulmqi city (Chinese) [Master]: Xinjiang Medical University; 2018.

[20] WU H, WANG J, LI W. Prevalence rate and influencing factors of hyperuricemia among middle-aged and elderly people in Akesu area of Xinjiang from 2014-2016 (Chinese). Occupation and Health. 2017;33(14):1952-1954+1962.

[21] HE Y. Analysis on the prevalence and risk factors of hyperuricemia among teaching and administrative staffs in universities in Dalian city (Chinese). Chinese Journal of School Health. 2017;38(10):1594-1596.

[22] LIU L, ZHAO J, ZHAO X, et al. Analysis of hyperuricemia prevalence and the related factors of university staff (Chinese). Journal of Northwest Minorities University (Natural Science). 2017;38(01):63-67.

[23] YAN X, WANG Z, SHANG H, et al. Investigation on metabolic diseases and its correlation among routine physical examination population in Jiayuguan city (Chinese). Gansu Medical Journal. 2017;36(10):881-883,888.

[24] BIAN W, Ge Y, CAO X, et al. The prevalence and correlation analysis of hyperuricemia among faculty in a university in Jiangsu Province (Chinese). Journal of Southeast University (Medical Science Edition). 2017;36(05):785-788.

[25] WANG J. Detection rate of hyperuricemia and its correlation with blood lipid level in Jinhua adult population (Chinese). Clinical Education of General Practice. 2017;15(01):71-73.

[26] JIANG C, LI X, LI Y, et al. Correlation analysis of blood uric acid level with gender and age among 3885 physical examinees in Kunming (Chinese). Journal of Modern Laboratory Medicine. 2017;38(05):671-673.

[27] KONG W. Investigation on the relationship between urolithiasis and hyperuricemia in Lianshan area (Chinese). Practical Clinical Journal of Integrated Traditional Chinese and Western Medicine. 2017;17(09):21-23.

[28] FENG J, CUI J, XU X, et al. Analysis of the health examinations results of people in the government and other institution (Chinese). Education Science Forum. 2017 (52):51-52.

[29] Guo X, Zhang Y. Analysis of serum uric acid in 1160 physical examinees in Ningxia Province (Chinese). Ningxia Medical Journal. 2017;39(9):840-842.

[30] CHEN X, LI N, CHEN Y, et al. Epidemiological studies of infected factors on gout and high uric acid hematic disease in Qinzhou district (Chinese). Laboratory Medicine and Clinic. 2017;14(23):3470-3472.

[31] LIU S, LAI H, HE N. Prevalence and risk factors of hyperuricemia among elderly population in a community of Shanghai (Chinese). Chinese Journal of Public Health. 2017;33(10):1445-1449.

[32] HAN J, CAI L. Analysis of the prevalence of hyperuricemia and its related risk factors in Weifang city (Chinese). Chinese Journal of Integrative Medicine on Cardio-/Cerebrovascular Disease. 2017;15(02):220-222.

[33] WANG F, LIU P, CHEN H, et al. Analysis of related factors of hyperuricemia in Kuitun district of Xinjiang (Chinese). Jilin Medical Journal. 2017;38(07):1292-1295.

[34] ZHANG W, KOU L, WANG H. Correlation between high blood uric acid and hyperlipidemia among railway workers in Yinchuan city (Chinese). Oriental Diet-Therapy and Health Care. 2017 (11):39,41.

[35] XIE Y, LUO R, SONG Y, et al. Prevalence and influence factors of hyperuricemia among residents in eastern mountain area of Guangdong province (Chinese). Chinese Journal of Public Health. 2017;33(02):317-320.

[36] HE X, DUAN X, HU X, et al. Study on prevalence and risk factors of hyperuricemia in Changsha city (Chinese). Shenzhen Journal of Integrated Traditional Chinese and Western Medicine. 2017;27(17):31-33.

[37] DAN S, CHENG K, HUANG J, et al. Analysis of hyperuricemia in 1276 physical examination population (Chinese). Journal of Diseases Monitor & Control. 2016;10(07):557-558.

[38] ZHANG X, ZHANG J. Analysis of serum uric acid levels and hyperuricaemia of 6719 healthy controls (Chinese). Journal of Shanxi Medical College for Continuing Education. 2016;26(03):17-20.

[39] WANG Y. Change on the prevalence of hyperuricemia between 2000 and 2009 in a community-based elderly population in Beijing (Chinese) [Master]: Capital Medical University; 2016.

[40] WANG Y. Change on the prevalence of hyperuricemia between 2000 and 2009 in a community-based elderly population in Beijing. (Chinese) [Master]: Capital Medical University; 2016.

[41] ZHANG W, WEI Q, GUO Z, et al. Analysis of blood uric acid level and risk factors in healthy population from Dongguan during physical examination (Chinese). New Medicine. 2016;47(03):162-165.

[42] LI H, LU H. Analysis of prevalence and risk factors of hyperuricemia in physical examination population in Guangzhou city (Chinese). The Journal of Medical Theory and Practice. 2016;29(20):3560-3561.

[43] SONG Z, DONG H, CHEN Z, et al. Prevalence of hyperuricemia in physical examination population from Tianhe District of Guangzhou (Chinese). New Medicine. 2016;47(03):195-198.

[44] XIE J. Analysis of hyperuricemia and related factors among 5020 airline staffs (Chinese). Journal of Aerospace Medicine. 2016;27(04):451-453.

[45] SHENG P. Analysis of hyperuricemia and associated metabolic syndrome in community residents (Chinese). Psychological Doctor. 2016;22(2):11-12.

[46] ZHANG J, LIU C, GENG W, et al. Epidemiological survey of hyperuricemia in young worker of Shengli oilfield Dongying (Chinese). Clinical Medicine. 2016:2.

[47] QUAN L, WANG J, YUWEN B, et al. Epidemiological survey of prevalence of hyperuricemia and its related factors in Uygur population (Chinese). Public Medical Forum Magazine. 2016;20(26):3710-3713.

[48] SHEN Y, ZHANG J, JIANG Y, et al. The prevalence of hyperuricemia and its influencing factors among the employees in a large state-owned enterprise of western China (Chinese). Chinese Journal of Disease Control & Prevention. 2016;20(10):991-994.

[49] LI Q, CHEN H, LU A, et al. Investigation on the prevalence of hyperuricemia among civil servants in Qijiang district of Chongqing city (Chinese). China Health Care Nutrition. 2016;26(12):305-306.

[50] LIU X, HAN Y, JIANG J. Survey on hyperuricemia and related diseases among university staffs in Guangdong in 2013 (Chinese). Chinese Journal of School Doctor. 2015;29(06):412-413.

[51] ZHOU M, KANG L, LIANG J. Investigation on epidemiological status of hyperuricemia on 5802 cases of railroaders (Chinese). Journal of Henan Medical College. 2015;27(01):63-64.

[52] JIANG F. Analysis of serum uric acid level in 51056 healthy subjects (Chinese). The Journal of Medical Theory and Practice. 2015;28(24):3409-3410.

[53] LV J. Survey on hyperuricemia in community of Anning city (Chinese). Journal of Dali University. 2015;14(10):49-51.

[54] ZHANG L, LIU C. Analysis of epidemic characteristics of hyperuricemia in people with medical examination in Baiyin city (Chinese). Bulletin of Disease Control & Prevention（China）. 2015;30(01):41-42+47.

[55] ZHOU F, MAO B, XUE F. Analysis on the prevalence of hyperuricemia among railway workers in Bengbu area (Chinese). Journal of Mathematical Medicine. 2015;28(02):224-225.

[56] FAN Y, JIANG M, CHENG Y, et al. Study on the prevalence of hyperuricemia and its correlation with blood pressure, blood glucose and blood lipid levels in elderly population in urban communities (Chinese). Journal of Ningxia Medical University. 2015;37(02):154-157.

[57] LI Y, FANG X, ZHONG G, et al. Prevalence of rural elderly hyperuricemia in Shijie, Dongguan and analusis of correlation between hyperuricemia and blood lipids (Chinese). Chinese Primary Health Care. 2015;29(04):60-62.

[58] LIN J. Incidence of hyperuricemia and related risk factors in 5381 cases in island area (Chinese). Chinese Journal of General Practice. 2015;13(03):509-511.

[59] ZHANG H, TAN X, DENG W, et al. Analysis on prevalence of hyperuricemia among urban population in Kaiping city (Chinese). Academic Journal of Guangzhou Medical University. 2015;43(04):111-113.

[60] ZHUO L, HUANG Y, HE W, et al. Serum level of uric acid and risk factors for hyperuricemia in elder population (Chinese). Jiangsu Medical Journal. 2015;41(02):146-148.

[61] LI R, LI W, WANG Y, et al. Analysis on prevalence and influence factors of hyperuricemia among residents in Liuzhou (Chinese). Chinese Journal of Health Laboratory Technology. 2015;25(16):2807-2809.

[62] LIU G, YIN J, HUANG J. Investigation on concentrations of blood uric acid of staff in a university (Chinese). Journal of Modern Laboratory Medicine. 2015;36(04):487-488+491.

[63] ZHU J. Investigation on the fasting serum uric acid level of 66 552 healthy checkup in Nanping city (Chinese). Chinese Journal of Health Laboratory Technology. 2015;25(13):2157-2159+2162.

[64] MEI H, LU L, QIAO P. Analysis on the prevalence rate of hyperuricemia and blood lipid level among physical examination population in Taiyuan city (Chinese). Shanxi Medical Journal. 2015;44(15):1755-1757.

[65] WU L, KUER B, SONG X, et al. The prevalence of hyperuricemia in Xinjiang Kazaks in Fuhai (Chinese). Chinese Journal of Internal Medicine. 2015;54(11):931-935.

[66] LUO C, YANG S, GONG F, et al. Epidemiological investigation of hyperuricemia in occupational population and its correlation with metabolic syndrome in Yichang city (Chinese). The Journal of Practical Medicine. 2015;31(19):3250-3253.

[67] DU L. Analysis of fhyperuricemia in college staff (Chinese). Health World. 2015;5(6):51-53.

[68] HAN Z. Longitudinal changes of GLU and UA among health check-up in 2006 and 2011 (Chinese). Journal of Southeast University（Medical Science Edition）. 2014;33(4):492-497.

[69] HAN Z. Longitudinal changes of GLU and UA among health check-up in 2006 and 2011. (Chinese). Journal of Southeast University（Medical Science Edition）. 2014;33(4):492-497.

[70] ZHANG G, CHEN H, YANG Z, et al. Investigation on the serum uric acid level and its relationship with hypertension and other related diseases among 13216 residents in island areas (Chinese). Practical Preventive Medicine. 2014;21(04):405-408.

[71] CUI D, LIU F, GONG J, et al. Epidemiological investigation of hyperuricemia among physical examination population in Baoding area (Chinese). Shandong Medical Journal. 2014;54(13):62-63.

[72] HAN Z, ZHANG L, HE J, et al. Analysis of the serum uric acid level in general population of Panyu district and relavant risk factors (Chinese). Hainan Medical Journal. 2014;25(24):3661-3664.

[73] YANG Y. Investigation and analysis of hyperuricemia and related diseases in college community (Chinese). Shaanxi Medical Journal. 2014;43(03):362-364.

[74] CHEN Y, FANG Z, TANG Z, et al. Investigation of hyperuricemia in residents of two regions in Guangxi province (Chinese). Practical Preventive Medicine. 2014;21(09):1133-1135+1128.

[75] SONG W, LIU J, CHEN Z, et al. Hyperuricemia and gout: a prevalence survey among over-40-year-old community residents in Nanchang district (Chinese). Chinese General Practice. 2014;17(02):181-184.

[76] QIANG Y, LIU H, SONG H, et al. The epidemiologic study of hyperuricemia among part of the populations in Ningxia (Chinese). Industrial Health and Occupational Diseases. 2014;40(06):423-426+432.

[77] CAO L, LIN H, ZHOU J, et al. The prevalence of hyperuricemia and its risk factors in the population of the outback of Shandong province (Chinese). Shandong University2014.

[78] XIE W, LIN Z, ZHANG X, et al. A survey on prevalence and risk factors of hyperuricemia in Shantou (Chinese). Journal of Clinical Nephrology. 2014;14(12):721-725.

[79] GAO N, XU N, LI X, et al. Analysis of prevalence and associated risk factors of hyperuricemia in subjects undergoing routine physical examinations in Xi’an pronvince (Chinese). Journal of Modern Laboratory Medicine. 2014;29(3):99-101,105.

[80] LI K, GESANG N, YANG X, et al. The current status of hyperuricemia between Tibetan and Han population on the Tibetan plateau (Chinese). Journal of High Altitude Medicine. 2014;24(03):29-32.

[81] ZHANG P, ZHANG L, WANG C, et al. Investigation of hyperuricemia and gout in people over 30 years old in Xingtai mountain area (Chinese). Practical Preventive Medicine. 2014;21(08):1010-1012.

[82] LIU M, HUANG Q, LI L, et al. The relationship between serum uric acid levels and metabolic diseases in elderly (Chinese). Journal of Nanjing Medical University(Natural Sciences). 2014;34(2):168-173.

[83] HUANG J, DONG L, ZOU G, et al. Epidemiological survey of hyperuricemia in the Guangxi urban community residents (Chinese). International Journal of Endocrinology and Metabolism. 2014;34(1):5-9.

[84] WANG E, LU X. Epidemiological analysis of uric acid level in adults of Changzhou city (Chinese) Occupation and Health. 2013;29(20):2675-2676.

[85] HAN T, LIU P, RONG S, et al. The prevalence of hyperuricemia in urban residents undergoing physical examination in Haerbing city (Chinese) Journal of Hygiene Research. 2013;42(02):306-307.

[86] ZHAO J. The survey and analysis of serum uric acid level in Fengfeng mining area (Chinese). Labeled Immunoassays and Clinical Medicine. 2013;20(03):151-153.

[87] CHEN X, LIU W, WANG N, et al. The prevalence and relevant factors analysis of hyperuricemia in Hui and Dongxiang pilgrims in Gansu province (Chinese). Chinese Journal of Prevention and Control of Chronic Non-Communicable Diseases. 2013;21(03):301-304.

[88] LIU Y. Analysis of uric acid in 47,904 physical examination subjects in Qinhuangdao city, Hebei province (Chinese). Shanxi Medical Journal. 2013;42(07):757-758.

[89] YANG F, HEN D, LI Q, et al. Analysis of the characteristics and related factors of hyperuricemia in elderly people of Jiading district (Chinese). Shanghai Medical & Pharmaceutical Journal. 2013;34(14):25-28.

[90] GUO Y. Analysis on the prevalence of hyperuricemia among teaching and administrative staffs in university (Chinese). Chinese Journal of School Health. 2013;34(1):124-125.

[91] SUN Z, WU Y, SUN H, et al. Analysis of prevalence of hyperuricemia among scientists and engineers in a scientific research institution (Chinese). Chinese Journal of General Practice. 2013;11(05):762-763.

[92] ZHAO Q, LI Z. Analysis of serum uric acid level in 12711 physical examinees in Nanchong area (Chinese). International Journal of Laboratory Medicine. 2013;34(24):3438-3439.

[93] JIA M, LU Y, WANG J, et al. Analysis on the prevalence and risk factors of hyperuricemia in 33,808 employees of Shijiazhuang city (Chinese). Chinese Journal of Difficult and Complicated Cases. 2013;12(03):229-231.

[94] QIAN D, Fan G, CHEN P, et al. Analysis of serum uric acid level and related risk factors in physical examination workers (Chinese). Chinese Journal of Cardiology. 2013 (01):60-64.

[95] DUAN W, ZHANG J, MA Y, et al. Prevalence and influencing factors of hyperuricemia among residents in Korla region of Xinjiang (Chinese). Chinese General Practice. 2013;16(10):916-918.

[96] WANG X. Epidemiological study on hyperuricemia in shayuan Street community, Haizhu district (Chinese). Uygur Medicine. 2013 (4):283-284.

[97] SONG A. Analysis of serum uric acid levels in 3424 patients over 60 years old (Chinese). Chinese Community Doctors. 2012;14(19):278.

[98] TAN Y, YANG B, LEI J, et al. Correlation analysis of hyperuricemia and the related diseases in parts of the elderly cadres in Chengdu city (Chinese) Hainan Medical Journal. 2012;23(19):99-101.

[99] LIU S, XU K, QIN K, et al. The prevalent characteristics and the analytical study of relevant factors of hyperuricemia in Chengdu (Chinese). Medical Journal of West China. 2012;24(03):474-476.

[100] WU X, MA X, CHEN Y, et al. Statistical analysis of uric acid level in 22995 physical examinees in Dongguan city (Chinese). Hainan Medical Journal. 2012;23(01):129-131.

[101] LU J, LIU Q, WANG M, et al. Study on the prevalence of hyperuricemia and metabolic syndrome in the senior of Guizhou (Chinese). Guizhou Medical Journal. 2012;36(02):115-118.

[102] HU X, XIAO C, WANG J. Investigation of hyperuricemia among workers in Haiyan county (Chinese). Zhejiang Journal of Preventive Medicine. 2012;24(07):43+45.

[103] MENG J, ZHU Y, TAN W, et al. Prevalence of hyperuricemia in rural residents of Gaoyou city, Jiangsu province (Chinese) Chinese Journal of Rheumatology. 2012;16(7):436-441.

[104] FENG J, ZHANG Z, LI Y. The investigation and analysis of blood uric acid levels and hyperuricemia in a college freshmen (Chinese). Journal of Kunming Medical College. 2012;33(03):93-95.

[105] YANG Z, CHEN H, HE Z, et al. Cross-sectional study on prevalence and risk factors of hyperuricemia among the residents in an oil field (Chinese). Practical Preventive Medicine. 2012;19(02):285-287.

[106] WANG M, FAN L, BAO Z. Relationship between hyperuricemia and related diseases in physical examination population (Chinese). Medical Journal of the Chinese People＇s Armed Police Forces. 2012;23(3):241-242.

[107] WU L, SONG X, KUER B, et al. Epidemiological study on hyperuricemia and gout in Uygur population in Turpan area of Xinjiang (Chinese). Journal of Peking University (Health Sciences). 2012;44(02):250-253.

[108] SHE D, ZHU Y, FENG S, et al. Investigation on the incidence and risk factors of hyperuricemia in Yangzhou (Chinese). Clinical Focus. 2012;27(05):417-419.

[109] LIU L, ZHAO J, PANG C, et al. Prevalence of hyperuricemia and influencing factors among civil servants in Zhengzhou (Chinese). Journal of Zhengzhou University（Medical Sciences）. 2012;47(03):390-393.

[110] FENG X. Analysis of hyperuricemia in health examination population in Diqing Tibetan Autonomous Prefecture (Chinese). China Health Care Nutrition. 2012 (4):393-394.

[111] WANG J, WANG L. Analysis of the prevalence and risk factors of hyperuricemia in healthy physical examination population (Chinese). China Health Care Nutrition. 2012;22(9):3153-3153.

[112] QIAN B, ZHANG Y. Analysis of chronic non-communicable diseases in some healthy people in Shanghai in 2010 (Chinese). Chinese Community Doctors. 2011;13(25):235-236.

[113] WEN X. Analysis of serum uric acid test in 5811 healthy people (Chinese). China Medicine and Pharmacy. 2011;1(15):157-158.

[114] HUANG F, DIAO M, CHEN X. Epidemiological investigation of serum uric acid level in 29234 physical examination subjects (Chinese). Chinese Journal of Convalescent Medicine. 2011;20(01):83-84.

[115] LIANG Q, ZHOU Y, YIN J, et al. Research about relationship between hyperuricemia and essential hypertension of employees at Guangzhou railroad area (Chinese). Journal of Taishan Medical College. 2011;32(01):55-58.

[116] YANG Y. Analysis on the prevalence and risk factors of hyperuricemia among university staffs in Lanzhou city (Chinese). Chinese Primary Health Care. 2011;25(04):98-99.

[117] LIU Q, WANG X, LU C, et al. Clinical analusis of hyperuricemia in civil servants from the city of Lianyungang city (Chinese). Medical Information. 2011;24(03):1208-1210.

[118] ZHANG C, XIE F. Study on the correlation between hyperuricemia and blood lipid and body mass index in 2420 middle-aged and elderly patients (Chinese). The Chinese and foreign health abstract. 2011;08(8):231-232.

[119] HUANG H, LIU R, CHEN J, et al. The analysis of blood uric acid level and the indicators associated with hyperuceIIlia in inhabitants in Beihai (Chinese). Acta Academiae Medicinae Xuzhou. 2011;31(1):48-50.

[120] CHEN J, LI S, OUYANG S, et al. Survey of hyperuricemia in middle-aged and elderly people in community and study on community nursing intervention for them (Chinese). Chinese Nursing Research. 2011;25(36):3313-3315.

[121] ZHU Q, SHI L, HUANG Z, et al. Analysis of hyperuricemia in health check-up population in Shenzhen city (Chinese). International Journal of Nursing. 2011;30(5):655-657.

[122] LIU J, YU H, HU L, et al. Analysis on the prevalence and related factors of hyperuricemia in 11,620 medical patients in a medical institution in Taiyuan city (Chinese). Chinese Journal of Rheumatology. 2011;15(3):209-211.

[123] WANG H, LIU Y, ZENG H, et al. Prevalence of hyperuricemia and the analysis of blood uric acid and creatinine in 5883 inhabitants (Chinese). Journal of Dalian Medical University. 2011;33(03):291-293.

[124] LIU L, ZHAO H, ZHNG Q, et al. Blood uric acid levels of routine health check-up receivers in Tianjin city (Chinese). Chinese Journal of Health Management. 2011;05(2):79-82.

[125] JIA Q, LOU S, MENG Z, et al. Hyperuricemia increases risk of metabolic syndrome in women (Chinese). Chinese Journal of General Practitioners. 2011;10(8):567-569.

[126] CHEN M, ZHANG X, ZHAO J, et al. Prevalence status and risk factors analysis of hyperuricacidem ia among physical examination samples of Xuzhou area (Chinese). Acta Academiae Medicinae Xuzhou. 2011;31(10):683-686.

[127] LI G. Blood uric acid detection and analysis of 1304 healthy population (Chinese). Nei Mongol Journal of Traditional Chinese Medicine. 2010;29(19):103-104.

[128] CHEN F. Kunming citizens′ hyperuricemia investigation in 2007 health examination (Chinese). Medical Recapitulate. 2010;16(05):797-798.

[129] FAN Q. The analysis of blood uric acid level and the indications associate with hyperuricemia in 5400 Beihai's inhabitants (Chinese) [Master]: Guangxi Medical University; 2010.

[130] JIANG Y. Analysis of serum uric acid level among health examinees in Beijing city (Chinese). Chinese Journal of Coal Industry Medicine. 2010;13(2):271-271.

[131] ZHENG J, CHEN P, XIE J. Analysis on prevalence rate and influential factors of hyperuricemia among residents in Ouhai district (Chinese). Zhejiang Journal of Preventive Medicine. 2010;22(12):13-16.

[132] HOU J, LUO L, CHENG L, et al. Analysis on prevalence and influence factors of hyperuricemia among residents in Dalian Zhangzidao (Chinese). Journal of Medical Research. 2010;39(02):37-40.

[133] ZHANG L. Analysis of the relationship between compositions of hyperuricemia and metabolic syndrome among university teachers (Chinese). Chongqing Medicine. 2010;39(03):311-312.

[134] Deng Z, WANG B. Epidemiological analysis of hyperuricemia among healthy physical examination population in Haikou city (Chinese). Journal of Third Military Medical University. 2010;32(24):2671-2672.

[135] ZHANG J, ZHANG P. Investigation of hyperuricemia in health examinees in Karamay district (Chinese). Chinese Community Doctors. 2010;12(22):206.

[136] YANG X, MA J, SHI L, et al. Investigation of hyperuricemia in the elderly over 60 years old in Beicai district, Shanghai (Chinese). Practical Journal of Cardiac Cerebral Pneumal and Vascular Disease. 2010;18(11):1597-1598.

[137] WAN P, GUAN G, LI J. Correlations between serum uric acid level and components of metabolic syndrome in the civil servants of Shanghai (Chinese). China Practical Medicine. 2010;05(31):3-5.

[138] ZHANG L, ZHOU Y. Epidemiological survey of gout and hyperuricemia among young and middle-aged people in Tangshan community (Chinese). Journal of Shandong University（Health Sciences）. 2010;48(05):163-164.

[139] WANG J, SHAO Y, CHEN Y, et al. Analysis on Wenzhou citizens' hyperuricemia and the influence factor (Chinese). Chinese Journal of Health Laboratory Technology. 2010;20(10):2545-2547.

[140] SHEN H, LU W, HUA J, et al. Analysis of the prevalence and related risk factors of hyperuricemia in 14,485 residents in Wuxi city (Chinese). Suzhou University Journal of Medical Science. 2010;30(03):582-583+618.

[141] CHEN X, LI K, JIANG X. Investigation on hyperuricemia in healthy population in underdeveloped areas of western China (Chinese). Chinese Journal of Clinical Research. 2010;23(06):549-550.

[142] LI S, YU J, LV S, et al. The prevalence and risk factors of hyperuricaemia in Yan′an city (Chinese). Chinese Preventive Medicine. 2010;11(08):763-765.

[143] ZHANG Q, ZHANG L, JIAO H, et al. Level of serum uric acid and correlation factors in young and middle aged population (Chinese). Clinical Focus. 2010;25(17):1501-1504.

[144] TAN L, REN J, HE H, et al. Survey the detection rate of hyperuricemia in physical examination personnel in Chongqing area (Chinese). International Journal of Laboratory Medicine. 2010;31(10):1101+1103.

[145] WANG L, ZHANG J. Investigation of uric acid concentration in healthy people in Shanxi area (Chinese). Chinese Community Doctors. 2009;11(22):258-259.

[146] WU H, YUAN P, RONG S, et al. Correlations between hyperuricemia and a variety of metabolic-related factors in cadre members in Jiangmen cty, Guangdong (Chinese). Chinese Journal of Prevention and Control of Chronic Diseases. 2009;17(02):174-176.

[147] LIU Q, LI S, ZHANG H. Analysis on the risk factors of hyperuricemia in Lishui city (Chinese). Shanghai Journal of Preventive Medicine. 2009;21(11):533-534.

[148] HUANG H, JIN H, CHEN Z. Analysis of serum uric acid in healthy subjectsamong Xiamen city (Chinese). Henan Journal of Preventive Medicine. 2009;20(05):345-346+350.

[149] JIA Y, CUI L, YANG W, et al. Epidemiological survey on morbidity pf hyperuricemia and gout in Tangshan mining district (Chinese). Chinese Journal of Coal Industry Medicine. 2009;12(12):1933-1935.

[150] LV C, PENG J, LIU X, et al. Investigation and analysis of hyperuricemia in han people in Urumqi city (Chinese). Internal Medicine. 2009;4(06):907-908.

[151] YANG C, LV G, LIU Y, et al. Hyperuricemia in Tibetans undergoing health examination in Linzhi district in Tiebet (Chinese). Military Medical Journal of South China. 2009;23(6):10-11,33.

[152] CHEN X, YANG H, YANG J. Prevalence of hyperuricemia and gout among residents in Dali city of Yunnan province (Chinese). China Practical Medicine. 2009;4(10):257-259.

[153] CAO L, HONG X. Analysis of serum uric acid level in a population of Zhenhai district (Chinese). Laboratory Medicine. 2009;24(11):804-807.

[154] GAO C, JIANG Y, TANG Z. Epidemiological analysis of hyperuricemia in 39824 healthy subjects in Anhui province (Chinese). The Journal of Practical Medicine. 2008 (20):3589-3590.

[155] CHEN C, ZHANG W, LIANG H, et al. The incidence and its related factor analysis of hyperuricema among chengdu railway employees (Chinese). Sichuan Medical Journal. 2008 (09):1139-1140.

[156] LIU M, YU Y, DONG F, et al. Examination and analysis of blood uric acid Level in 2, 067 inhabitants of Dalian (Chinese). Practical Preventive Medicine. 2008 (05):1602-1604.

[157] ZENG Y, HUANG X, ZHOU G, et al. The prevalence and related factors of hyperuricemia among 2340 public servants in Changsha city (Chinese). Journal of Hygiene Research. 2008 (06):679-681.

[158] WU W, GUO J, YANG W, et al. Epidemiology of hyperuricemia and gout in a community in Guangzhou (Chinese). Chinese Journal of General Practice. 2008 (07):728-729.

[159] WANG J, JIN J, LI C. Investigation and analysis of hyperuricemia in Jiamusi population (Chinese). Modern Chinese Doctor. 2008 (23):77-78.

[160] WEN X, LU F, YANG J, et al. Analysis on hyperuricemia and its influential factors in population (Chinese). Chinese Journal of Public Health. 2007 (12):1520-1522.

[161] DENG W, ZHANG Q, WANG X, et al. The current conditions of serum uric acid concentrations in parts of the middle-aged and elderly population of Shenyang (Chinese). Journal of China Medical University. 2007 (06):712-714.

[162] LI M. Comparative study on serum uric acid levels between Han and Korean residents in Yanbian area (Chinese) [Master]: Yanbian University; 2007.

[163] MIAO Z, ZHAO S, WANG Y, et al. Epidemic characteristics of gout and primary hyperuricemia in Shandong coastal area: a randomized stratified cluster sampling survey (Chinese). Chinese Journal of Tissue Engineering Research. 2007 (30):6087-6091.

[164] YAO Z, JIANG S, LIU H, et al. Epidemiological study on hyperuricemia and gout in the coastal area of Qingdao city (Chinese) Chinese Journal of Rheumatology. 2007;11(11):672-675.

[165] HUANG R, SONG X. Serum uric acid level and its relationship with hypertension in 6,687 government officials (Chinese). Chinese Journal of Convalescent Medicine. 2006 (06):404-406.

[166] FANG W, HUANG X, WANG Y, et al. A cross-sectional study of hyperuricemia in state-employees in Beijing: prevalence and risk factors (Chinese). National Medical Journal of China. 2006 (25):1764-1768.

[167] MAO Y, ZHOU L, YE H, et al. Epidemiological survey on prevalences of hyperuricemia and gout in staff of a petrochemical corporation in Ningbo Chinese). Chinese Journal of Endocrinology and Metabolism. 2006 (04):338-341.

[168] YU J, LU J, ZHANG X, et al. The analysis of blood uric acid level and the indications associate with hyperuricemia in 13 324 Foshan's inhabitants (Chinese). Chinese Journal of Integrated Traditional and Western Nephrology. 2005 (07):401-403.

[169] ZENG X. Epidemiological survey on hyperuricemia and gout among railway workers in Nanning (Chinese). Heilongjiang Medical Journal. 2005 (11):78-79.

[170] YANG Y, LIU X, XIE H, et al. Association between prevalence rate of hyperuricemia and risk factors of cardiovascular disease in the population of Qingdao port (Chinese). Chinese Journal of Clinical Rehabilitation. 2005 (43):1-3.

[171] SHAO J, MO B, YU R, et al. Epidemiological study on hyperuricemia and gout in community of Nanjing (Chinese). Chinese Journal of Disease Control & Prevention. 2003 (04):305-308.

[172] Qiu L, Cheng XQ, Wu J, et al. Prevalence of hyperuricemia and its related risk factors in healthy adults from Northern and Northeastern Chinese provinces. BMC public health. 2013 Jul 17;13:664.

[173] Liu L, Lou S, Xu K, et al. Relationship between lifestyle choices and hyperuricemia in Chinese men and women. Clinical rheumatology. 2013 Feb;32(2):233-9.

[174] Guo M, Niu JY, Li SR, et al. Gender differences in the association between hyperuricemia and diabetic kidney disease in community elderly patients. Journal of diabetes and its complications. 2015 Nov-Dec;29(8):1042-9.

[175] Shen Y, Wang Y, Chang C, et al. Prevalence and risk factors associated with hyperuricemia among working population at high altitudes: a cross-sectional study in Western China. Clinical rheumatology. 2019 May;38(5):1375-1384.

[176] Liu H, Zhang X-M, Wang Y-L, et al. Prevalence of hyperuricemia among Chinese adults: A national cross-sectional survey using multistage, stratified sampling. Journal of nephrology. 2014 04/01;27.

[177] Fan J, Li H, Liu X, et al. The prevalence of obesity and metabolic abnormalities in eastern China: a cross-sectional study. International Journal of Diabetes in Developing Countries. 2019 03/11;39.
